# BACH2 alleviates immune checkpoint inhibitors‐induced cardiac pyroptosis via transcriptionally promoting GRSF1

**DOI:** 10.1002/ctm2.70618

**Published:** 2026-02-10

**Authors:** Mengying Cao, Zilong Liu, Di Zhao, Hongyuan Zhang, Xinjie He, Wenhao Wang, Xiaolin Wang, Cheng Yang, Pan Gao, Yunzeng Zou

**Affiliations:** ^1^ Shanghai Institute of Cardiovascular Diseases and State Key Laboratory of Cardiovascular Diseases Zhongshan Hospital and Institutes of Biomedical Sciences Fudan University Shanghai China; ^2^ Department of Respiratory Medicine Zhongshan Hospital Shanghai Medical College of Fudan University Shanghai China; ^3^ Department of Cardiac Surgery Shanghai Institute of Cardiovascular Diseases Zhongshan Hospital, Fudan University Shanghai China; ^4^ Institutes of Advanced Medical Sciences and Huaihe Hospital Henan University Kaifeng China; ^5^ Qing Pu Branch of Zhongshan Hospital Fudan University Shanghai China

**Keywords:** BACH2, immune checkpoint inhibitors, lipoic acid, pyroptosis, subclinical myocardial stress

## Abstract

**Background:**

Immunotherapy has revolutionized the treatment of malignant tumors; however, it may lead to fatal cardiotoxicity. Herein, we explored the mechanisms underlying cardiac side‐effects induced by immune checkpoint inhibitors (ICIs) and proposed a promising therapeutic target.

**Methods:**

Serum samples were collected from 168 patients with advanced non‐small cell lung cancer (NSCLC) receiving ICIs treatment or not. Representative ICI (IBI308) was intraperitoneally injected into normal C57BL/6 and congenital immune deficient nude mice. NOD‐like receptor family, pyrin domain containing 3 (*Nlrp3*) globally knockout mice and gasdermin D (*Gsdmd*) globally knockout mice were involved in this study. Mice with cardiac‐specific BTB domain and CNC homolog 2 (Bach2) knock‐in and knock‐out were also included. The Cleavage Under Targets and Tagmentation (CUT&Tag) experiment was conducted to identify downstream molecules of BACH2, which was further validated with dual‐luciferase and electrophoretic mobility shift assays (EMSA). A library of small‐molecule products was screened to identify a specific agonist of BACH2, followed by in vivo and in vitro verification.

**Results:**

Patients treated with ICIs had significantly higher cardiac troponin T (cTNT) and interleukin 18 (IL‐18) levels. IBI308 significantly reduced cardiac function, increased cardiac fibrosis, and induced myocyte pyroptosis in wild type mice and T‐cell deficient nude mice. IBI308‐elicited toxicity was reversed by depleting pyroptotic genes *Nlrp3* or *Gsdmd*. Furthermore, cardiac‐specific knock‐in of Bach2 rescued, whereas cardiac‐specific knock‐out of Bach2 exacerbated IBI308‐induced cardiotoxicity and pyroptosis. BACH2 directly bound to the promoter of G‐Rich RNA sequence binding factor 1 (*GRSF1*) and promoted its transcription, which then activated the nuclear factor κB (NF‐κB) signaling cascade. The protective effect of BACH2 was dismissed after knockdown of *GRSF1* or inhibition of the NF‐κB pathway. Lipoic acid was identified as an activator of BACH2 and reversed IBI308‐induced pyroptosis in a BACH2‐dependent manner.

**Conclusions:**

ICIs treatments caused preclinical cardiac injuries by activating myocyte pyroptosis. BACH2 exerted protective effects by promoting *GRSF1* transcription and suppressing pyroptosis. Lipoic acid attenuated ICI‐induced cardiotoxicity by upregulating BACH2, which might be a novel therapeutic strategy.

**Key points:**

- Immune checkpoint inhibitors cause elevated cardiac injuries in humans and mice
- ICIs cause myocyte pyroptosis and cardiotoxicity not via the adaptive immune system
- BACH2 ameliorates ICIs‐induced pyroptosis through transcriptionally promoting *GRSF1*.
- Lipoic acid as a transcriptional inducer of BACH2 suppresses ICIs‐induced cardiotoxicity

## Graphical abstract

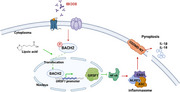

## 1 INTRODUCTION

Over the past decade, immunotherapy has substantially improved the prognosis of many malignant tumours. Inhibitors targeting programmed death receptor 1/ programmed death ligand 1 (PD‐1/PD‐L1), cytotoxic T lymphocyte‐associated antigen 4 (CTLA‐4), killer group 2 member A (NKG2A), killer cell immunoglobulin‐like receptors (KIRs), and T cell immune receptor with Ig and ITIM domains (TIGIT) have demonstrated remarkable advancements.^1^ However, immunotherapy also unexpectedly induces peripheral immune tolerance and prevents autoimmune responses. Therefore, immunotherapy such as immune checkpoint inhibitors (ICIs) treatment‐associated immune‐related adverse events (irAEs) have represented great clinical concerns.^2^, ^3^, ^4^ Cardiac irAEs manifest in various forms, including myocarditis,^5^, ^6^ pericarditis,^7^ arrhythmia, heart failure,^8^ and Takotsubo cardiomyopathy,^9^ with severity ranging from mild discomfort to sudden cardiac death.^6^ Although glucocorticoids, immunosuppressors,^10^ immunoglobulin, plasmapheresis and immunoadsorption therapies are effective regimens to relieve irAEs in some patients, the mortality of ICIs‐related cardiotoxicity remains high.^11^ As most oncology trials do not routinely monitor cardiac function and myocardial markers, the actual incidence of ICIs‐induced cardiotoxicity remains unclarified^12^ and may be even higher among older patients or those with prior cardiovascular diseases. Combined or sequential therapy of ICIs with radiotherapy, chemotherapy, and anti‐angiogenic drugs may also increase adverse cardiovascular events. Therefore, cardiotoxicity monitoring and pathogenesis exploration are urgently needed to propose effective treatments.

Mechanisms underlying irAEs include T‐cell‐mediated multiple organ damage, autoantibody abnormalities, monocyte‐driven inflammatory cascades and cytokine dysregulation.^13^ While the detailed injury types vary across models, cardiac inflammatory injury is the shared basis for ICIs treatment‐associated irAEs. Many reports have attributed the immune cell disturbance to the ICIs’ cardiotoxicity. For example, autoimmune T cells recognizing cardiac myosin,^14^ B cells producing cardiac autoantibodies,^15^ and inflammatory macrophages infiltration^16^ have been shown to be involved in the ICIs‐induced cardiotoxicity. However, it remains unaddressed whether ICIs could elicit cardiotoxic effects directly. Many confounding factors contributed to this debate, including unstable mouse models, different cardiotoxic stages and incomplete measurements.

As one of the cell inflammatory death forms, pyroptosis is a caspase‐dependent, gasdermin D (GSDMD)‐mediated programmed cell death characterized by cell swelling, membrane rupture, chromatin condensation, and nuclear integration. It can affect various cell types, including cardiomyocytes,^17^, ^18^, ^19^ cardiac fibroblasts,^20^, ^21^ vascular endothelial cells (VECs),^22^, ^23^, ^24^ vascular smooth muscle cells (VSMCs),^25^ and macrophages.^26^ Pyroptosis is closely related to a series of cardiovascular diseases, including atherosclerosis,^27^ myocardial ischemia‐reperfusion injury,^28^, ^29^ arrhythmia,^30^ myocarditis,^19^, ^31^ diabetic cardiomyopathy,^17^ and drug cardiotoxicity.^32^, ^33^, ^34^ However, the involvement of pyroptosis, either directly or depending on immune cells, in ICIs’ cardiotoxicity remains unclear.

The BTB domain and CNC homolog 2 (BACH2) is a transcription factor with a basic leucine‐zipper structure.^35^ BACH2 has been well established to participate in the regulation of immune cells and immune homeostasis,^36^ and is also associated with the sensitivity to ICIs treatment.^37^ Our group has supplied data showing that cardiomyocyte‐restricted BACH2 significantly attenuates cardiac hypertrophy^38^ and diabetic heart injury.^39^ Therefore, we further explore the detailed mechanism associated with BACH2 and the cardiac adverse events of ICIs.

This study aimed to systemically explore the ICIs‐elicited effects on cardiac function both in vivo and in vitro. We started by collecting clinical patients and tried to find any direct effects of ICIs treatments on the heart. We constructed mouse models that mimic the ICIs‐associated mild injury stage, and identified the predominant cell death types to depict the molecular events preceding the development of obvious lesions. We then focused on investigating the role of BACH2 in IBI308‐induced heart dysfunction by constructing both cardiomyocyte‐specific knockout (cKO) and transgenic (Tg) mouse strains. Furthermore, we also screened potential small‐molecule drugs that might serve as a specific agonist of BACH2 in attempts at clinical translation.

## 2 RESULTS

### 2.1 ICIs’ treatments associated with elevated cardiac injuries in clinical patients

**FIGURE 1:**
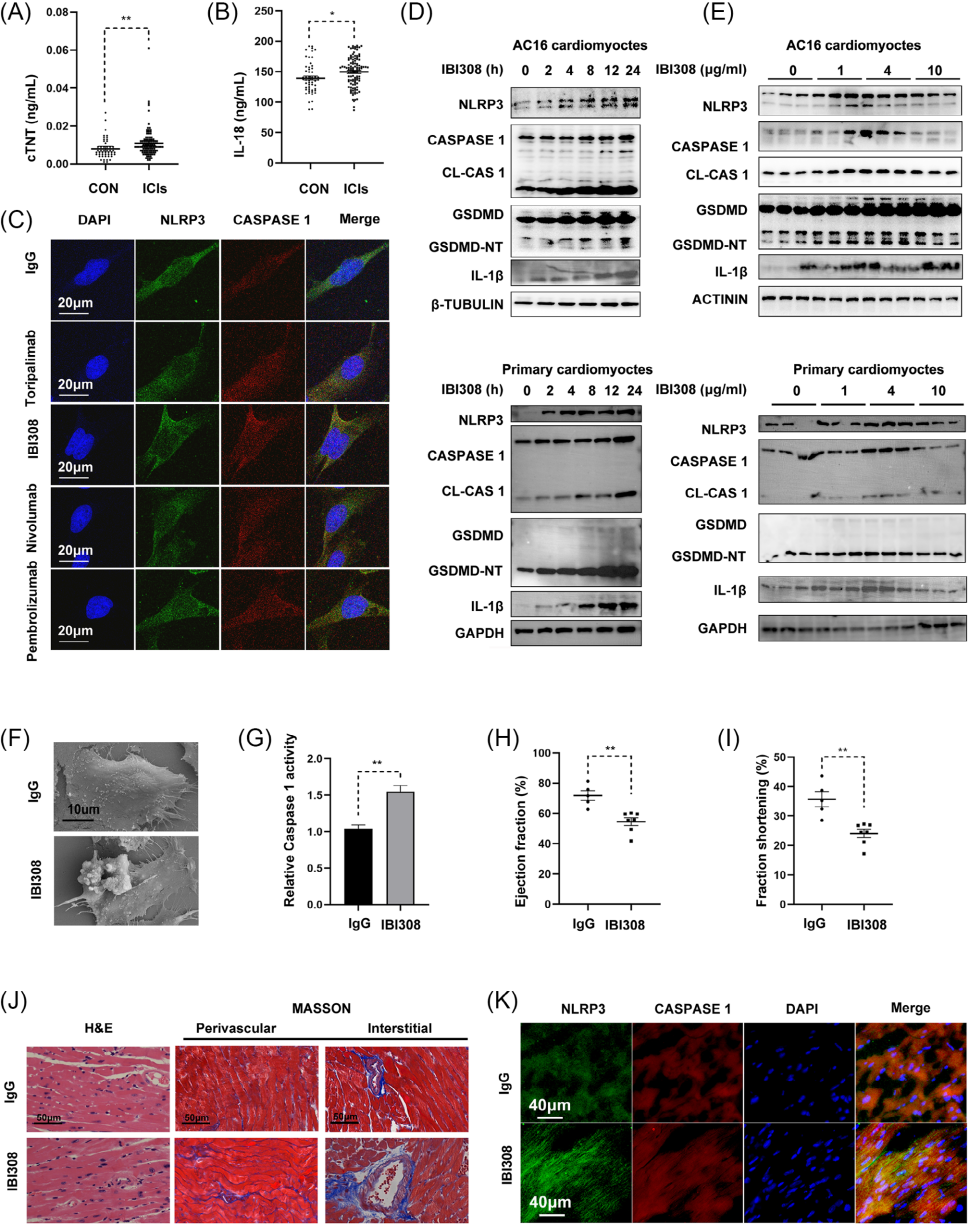
ICIs cause myocyte pyroptosis and cardiotoxicity. (A) The serum levels of cTNT in non‐small‐cell lung cancer (NSCLC) patients receiving (ICIs group, *n* = 111) or not receiving ICIs treatment (control or Con, *n* = 55). (B) ELISA analysis of serum IL‐18 levels in NSCLC patients with (*n* = 111) or without (*n* = 55) ICIs treatments. (C) Immunofluorescence analysis of NLRP3 (green), Caspase 1 (red) and their merged signals (yellow) in AC16 myocytes incubated with or without four ICIs for 24 h. Scale bar = 20 µm. (D) Time‐lapse development of pyroptosis in AC16 cardiomyocytes and primary cardiomyocytes treated with IBI308 (4 µg/µL). (E) Western blot analysis of pyroptosis proteins in AC16 cardiomyocytes and primary cardiomyocytes treated with IBI308 at the indicated doses for 24 h. (F) Representative scanning electron microscope (SEM) images of AC16 myocytes incubated with IBI308 (4 µg/µL) for 24 h. Scale bar = 10 µm. (G) Relative Caspase 1 activity in AC16 myocytes treated with or without IBI308 (4 µg/µL) for 24 h. (H, I) Ejection fraction and fraction of shortening in mice *i.p*. injected with IBI308 (50 mg/kg, weekly for a month) or vehicle (*n* = 5–7 per group). (J) HE staining and Masson Trichrome staining of the heart tissue in each mouse. Scale bar = 50 µm. (K) Immunofluorescence analysis of NLRP3 (green), Caspase 1 (red) and their merged signals (yellow) in mouse hearts with or without IBI308 treatment (50 mg/kg, weekly for a month). Scale bar = 40 µm. *p < .05; **p < .01 as indicated.

We collected 166 patients diagnosed with non‐small‐cell lung cancer (NSCLC)who were admitted to our hospital. Among these patients, 111 received ICIs treatments (ICIs group), while the remaining 55 patients (Control) were free from any ICI treatment during the period. As shown in Table 1, no significant differences were observed in age, sex, smoking habits, pathological types, TNM stages, surgery, radiotherapy, use of chemotherapies (including platinum, paclitaxel, pemetrexed, gemcitabine), angiogenesis inhibitors, and EGFR‐targeted drugs between the two groups. In the control group, seven patients were administered ALK/ROS‐targeted drugs, while no patients in the ICIs group received such treatment. However, patients in the ICIs group had significantly higher levels of cardiac troponin T (cTNT) (Figure 1A) and interleukin 18 (IL‐18) (Figure 1B). N‐terminal pro‐brain natriuretic peptide (NT‐proBNP) (Figure S1A) and IL‐1β (Figure S1B) were higher in the ICIs group. The proportion of NSCLC patients comorbid with elevated myocardial enzyme levels and/or electrocardiograph (ECG) abnormalities was also significantly higher in the ICIs group as compared with control patients (Figure S1C). These data indicated that ICIs treatments were associated with cardiac inflammatory injuries.

**TABLE 1:** Clinical characteristic of human samples.

	Control (*n* = 55)	ICIs (*n* = 111)	*p*‐value
Age (years)	60.7 ± 1.3	63.0 ± 0.9	.154
Sex (male%)	44(80.0%)	91(82.0%)	.758
Smoking [*n* (%)]	3 (5.5%)	4 (3.6%)	.768
Hypertension [*n* (%)]	18 (32.7%)	35 (31.5%)	.876
Pathology			
Adenocarcinoma [*n* (%)]	41 (74.5%)	71 (64.0%)	.171
SCC [*n* (%)]	11 (20.0%)	38 (34.2%)	.058
Other [*n* (%)]	4 (7.3%)	4 (3.6%)	.299
TNM Stage			.586
3A [*n* (%)]	6 (10.9%)	11 (15.3%)	
3B [*n* (%)]	9 (16.4%)	12 (10.8%)	
3C [*n* (%)]	6 (10.9%)	11 (9.9%)	
4A [*n* (%)]	19 (34.5%)	31 (27.9%)	
4B [*n* (%)]	15 (27.3%)	40 (36.0%)	
PS			.024*
0 [*n* (%)]	45 (81.8%)	72 (64.9%)	
1 [*n* (%)]	10 (18.2%)	39 (35.1%)	
Surgery [*n* (%)]	12 (21.8%)	24 (21.6%)	.977
Radiation [*n* (%)]	10 (18.2%)	19 (17.1%)	.865
Chemotherapy			
Platinum [*n* (%)]	13 (23.6%)	31 (27.9%)	.555
Paclitaxel [*n* (%)]	10 (18.2%)	22 (19.8%)	.801
Pemetrexed [*n* (%)]	16 (29.1%)	28 (25.2%)	.595
Gemcitabine [*n* (%)]	0 (0.0%)	5 (4.5%)	.171
Angiogenesis inhibitors [*n* (%)]	7 (12.7%)	10 (9.0%)	.457
Targeted			
EGFR [*n* (%)]	15 (27.3%)	19 (17.1%)	.127
ALK/ROS [*n* (%)]	7 (12.7%)	0 (0.0%)	0.000*
ICIs			
Anti‐PD‐1 [*n* (%)]	/	108 (97.3%)	/
Anti‐PD‐L1 [*n* (%)]	/	3 (2.7%)	/
Anti‐CTLA4 [*n* (%)]	/	1 (0.9%)	/
CEA	3.8 (2.2,12.0)	3.6 (2.3,11.6)	.898
CA125	18.0 (10.0,36.6)	18.4 (12.5,37.9)	.472
CA199	13.1(7.0,28.8)	13.2(8.0,25.9)	.751
NSE	13.8 (11.8,19.6)	14.6 (12.1,16.8)	.038*
Cyfra211	2.5 (1.7,4.0)	2.6 (1.7,4.5)	.089
SCCA	1.2 (0.9,2.0)	1.4 (1.0,2.2)	.530
Haemoglobin (g/L)	131.4 ± 2.0	127.7 ± 1.7	.188
WBC	5.5 (4.6,6.9)	5.9 (4.9,7.6)	.048*
NEUT (%)	64.1 ± 1.5	64.9 ± 0.9	.592
platelet	217.5 ± 8.0	263.5 ± 26.5	.231
TBIL	8.1 (5.9,12.9)	6.9 (5.3,8.4)	.000*
Alb (g/L)	45.0 (43.0,46.5)	44.0 (41.0,46.0)	.043*
eGFR (mL/min/1.73m^2^)	83.7 ± 2.2	80.0 ± 1.7	.102
cTNT (ng/mL)	0.007(0.005,0.009)	0.009(0.007,0.012)	.008*
NT‐proBNP (pg/mL)	35.0(15.5,58.6)	48.4(30.8,99.9)	.222
CK‐MB (U/L)	17.5 (14.0,18.8)	14.0 (11.0,18.0)	.969
CK‐MM (U/L)	35.0 (21.0,69.0)	62.0 (31.0,87.0)	.496
ECG	6 (10.9%)	17 (15.3%)	.511
IL‐1β	34.5 ± 0.9	34.9 ± 0.7	.758
IL‐18	139.5 ± 3.5	148.4 ± 2.6	.049*
LDH	42.8 ± 0.9	43.4 ± 1.2	.708
CTGF	2767.5 ± 56.3	2870.6 ± 42.9	.158
TGF‐β1	1067.0 ± 31.1	1088.4 ± 24.6	.606

Abbreviations: Alb, albumin; CEA, carcinoma embryonic antigen; ECG, electrocardiograph; EGFR, epidermal growth factor receptor; NSE, neuron‐specific enolase; PS, performance status; SCC, squamous cell carcinoma; SCCA, squamous cell carcinoma antigen; TBIL, serum total bilirubin.

^*^ *p* < .05, ICIs vs. Control.

### 2.2 ICIs directly cause myocyte pyroptosis and cardiotoxicity

To investigate the potential mechanisms underlying ICIs‐induced cardiac injuries, we used four ICIs, including Toripalimab, IBI308, Nivolumab and Pembrolizumab, to treat cardiomyocytes at a clinically comparable dose. We found that cell pyroptosis was remarkably activated in all drug‐exposed cells. In detail, all four ICI drugs promoted the formation of NLRP3 inflammasome (yellow signal) in the immunofluorescence assays (Figure 1C). In accordance, the protein levels of NLRP3, cleaved CASPASE‐1, GSDMD, IL‐1β and IL‐18 in AC16 cardiomyocytes and primary cardiomyocytes were all increased by the ICI treatments (Figure S1D). These observations suggested that ICI drugs may share some potential similar mechanisms to induce myocyte injury. We thus used IBI308 as a representative drug in view of its highest potential to evaluate cell pyroptosis and subclinical myocardial stress among the four ICIs. IBI308 directly reduced myocyte viability (Figure S1E) and increased the expression of pyroptosis markers in a dose‐ and time‐dependent manner in AC16 cells and primary cardiomyocytes (Figure 1D,E). Scanning electron microscopy (SEM) revealed that after 24 h IBI308 treatment, AC16 myocytes detached and displayed multiple bubble‐like protrusions, while control cells remained flattened and tightly attached to the culture slide (Figure 1F). Cell viability assays showed IBI308 caused cell death in a dose‐dependent manner (Figure S1E), as a reflection of myocyte pyroptosis, activity of caspase 1 was also notably increased upon IBI308 stimulation, indicating cell pyroptosis was involved in ICIs‐induced cardiotoxicity (Figure 1G). Next, we tried to examine IBI308 cardiotoxicity in vivo. To this end, we *i.p*. injected IBI308 into C57BL/6 mice and found IBI308 significantly reduced ejection fraction (EF) (Figure 1H) and fractional shortening (FS) (Figure 1I), showing direct irAEs in heart tissues. Masson Trichrome staining revealed a mild increase in cardiac fibrosis following IBI308 treatment (Figure 1J; Figure S1F). Moreover, IBI308‐treated hearts exhibited higher fluorescence intensity of NLRP3 (green) and CASPASE 1 (red) as well as their merged signals (yellow, denotes NLRP3 inflammasome) (Figure 1K), indicating the formation of NLRP3 inflammasome at the mild injury stage. We also examined other programmed cell death (PCD) pathways besides cell pyroptosis since various PCDs might be involved during cardiac injuries. As shown in Figure S1G–K, representative protein markers of cell apoptosis, necroptosis, autophagy and ferroptosis were not remarkably altered upon IBI308 treatment; however, cardiac pyroptosis markers, including NLRP3, CASPASE 1, GSDMD, IL‐1β, and IL‐18, were all dramatically increased in the IBI308‐exposed hearts, further indicating cell pyroptosis was the most obvious PCDs in ICIs‐induced cardiotoxicity.

### 2.3 Depletion of cell pyroptosis dismissed ICIs‐induced cardiotoxicity

To further assess whether pyroptosis is a causal factor for ICIs‐induced early cardiotoxicity, we constructed mouse models with genetic depletion of the pyroptosis initiation gene *Nlrp3* (*Nlrp3*‐KO) or effector gene *Gsdmd* (*Gsdmd*‐KO). Compared with wild‐type (WT) mice, both *Nlrp3*‐KO and *Gsdmd*‐KO mice showed resistance to IBI308, as evidenced by the improved cardiac functions (Figure S2A–F). IBI308 caused cardiac fibrosis and myocyte pyroptosis in WT mice, but not in *Gsdmd*‐KO mice (Figure S2G–I) or *Nlrp3*‐KO mice (Figure S2J–L). These results suggest that cardiac pyroptosis contributes to early ICIs cardiotoxicity.

### 2.4 ICIs directly caused myocyte pyroptosis and cardiotoxicity independent of PD‐1

IBI308 is a humanized PD‐1 inhibitor that has low affinity for mouse PD‐1. In view that IBI308 caused pyroptosis and cardiotoxicity in mouse hearts, we speculated that the side effects of IBI308 in mice may not depend on the presence of PD‐1. To verify this hypothesis, we constructed a *Pd‐1*‐KO mouse strain and i.p. injected IBI308 for 4 consecutive weeks. It turned out that even without PD‐1, IBI308 still induced cardiac dysfunction, myocardial fibrosis, and cardiomyocyte pyroptosis (Figure S3A–G). We further testified this in vitro with CRISPR/Cas9‐mediated *PD‐1*‐KO cells, IBI308 and other representative ICIs, including Toripalimab, Nivolumab, and Pembrolizumab, still increased pyroptosis markers, including NLRP3, CASPASE 1, GSDMD, and IL‐1β (Figure S3H,I).

Moreover, a humanized *PD‐1* (*hPD‐1*) mouse strain was also included to further verify our observations. As shown in Figure S4A–C, IBI308 injection caused cardiac dysfunction in WT‐type mice, as well as in *hPD‐1* mice. Histological analysis also elucidated that IBI308‐elicited cardiotoxic effects were proven in both *hPD‐1* mice and non‐*hPD‐1* mice (Figure S4D–F). Cell pyroptosis maker proteins remained largely unaltered in both groups (Figure S4G). Collectively, these data suggested ICIs directly caused myocyte pyroptosis and induced cardiotoxicity independent of PD‐1.

### 2.5 IBI308 induces cardiotoxicity by inhibiting the nuclear translocation of BACH2

**FIGURE 2:**
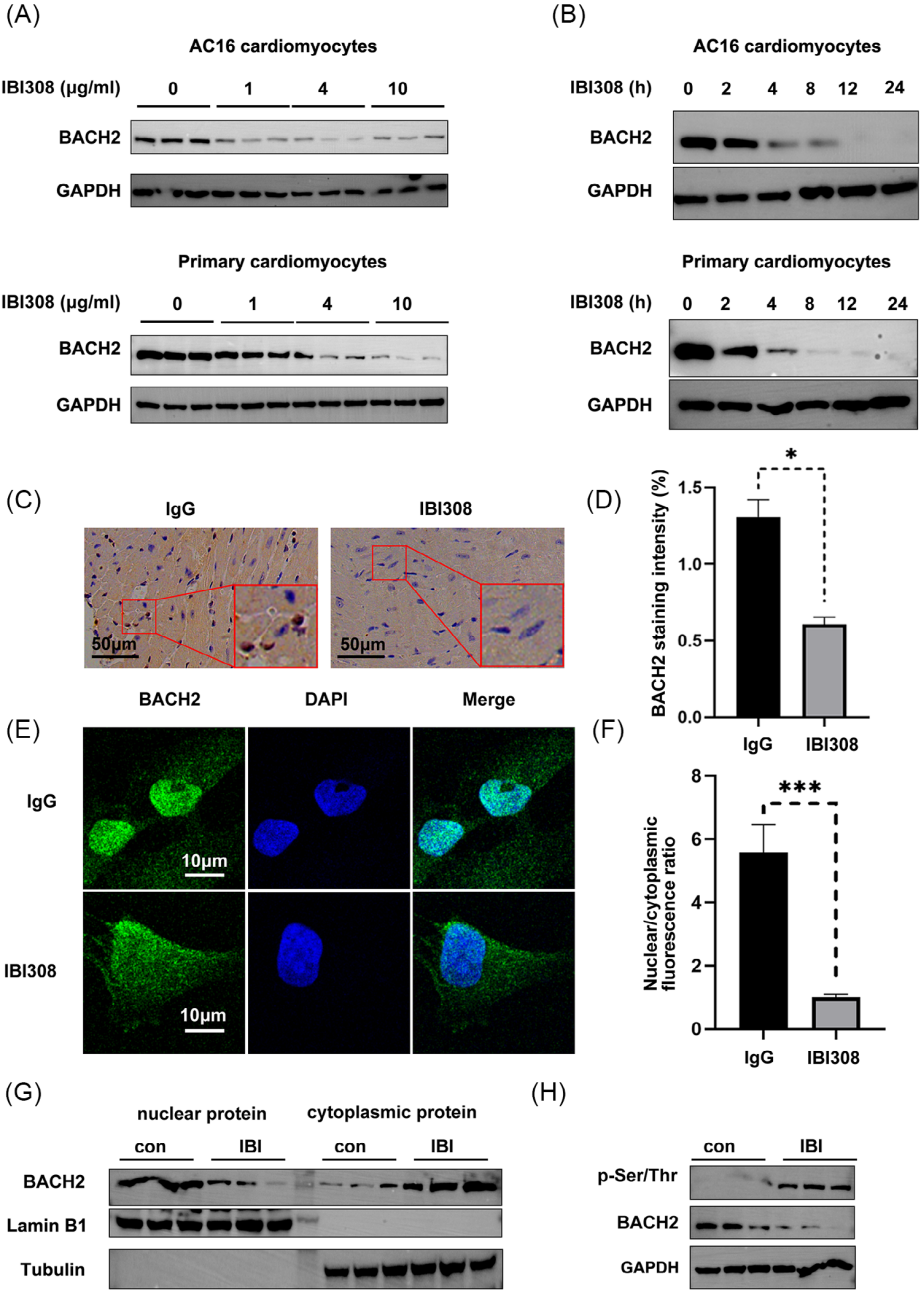
IBI308 inhibits BACH2 expression and prevents its nuclear translocation via enhancing its phosphorylation. (A) Western blot analysis of BACH2 in AC16 cardiomyocytes and primary cardiomyocytes treated with IBI308 for 24 h. (B) Western blot analysis of BACH2 in AC16 cardiomyocytes and primary cardiomyocytes treated with IBI308 (4 µg/µL) for different hours. (C) Immunohistochemistry staining of BACH2 in mouse hearts treated with IgG or IBI308 (50 mg/kg, weekly for a month). Scale bar = 50 µm. (D) Quantification of BACH2 staining intensity. (E) Immunofluorescence staining of BACH2 in AC16 myocytes treated with IgG or IBI308 (4 µg/µL). Scale bar = 10 µm. (F) Quantification of nuclear and cytoplasmic BACH2 immunofluorescence staining intensity. (G) Detection of nuclear and cytoplasmic BACH2 in AC16 myocytes treated with IgG or IBI308 (4 µg/µL) for 24 h. (H) The BACH2 antibody‐precipitated lysates were subjected to western blot analysis of the phosphorylation level of BACH2 in AC16 myocytes treated with IgG or IBI308 (4 µg/µL) for 24 h. *p < .05; ***p < .001; as indicated.

To identify core factors protecting against ICIs cardiotoxicity, we put particular interest into transcription factor BACH2, which is an immune regulatory factor^35^ and was previously revealed by us to maintain heart function in diabetic cardiomyopathy.^39^ It was shown that IBI308 treatment decreased the protein level of BACH2 in AC16 cardiomyocytes and primary cardiomyocytes in a dose‐ and time‐dependent manner (Figure 2A,B). In addition to expression decreases, IBI308 also inhibited Bach2 nucleus‐location in mouse hearts (Figure 2C,D) and in cultured AC16 myocytes (Figure 2E–G). As a transcription factor, the nuclear localization of BACH2 is crucial for its transcriptional activity, while phosphorylation of BACH2 limits its cytoplasm‐nucleus trafficking.^40^ We found that IBI308 increased the phosphorylation of BACH2 (Figure 2H), thereby maintaining its cytoplasmic location, which was accompanied by its protein level decline. These data revealed that IBI308 dismissed BACH2 function by promoting its phosphorylation and inhibiting its nuclear location.

### 2.6 BACH2 is a protective factor against ICIs‐induced cardiotoxicity and pyroptosis

**FIGURE 3:**
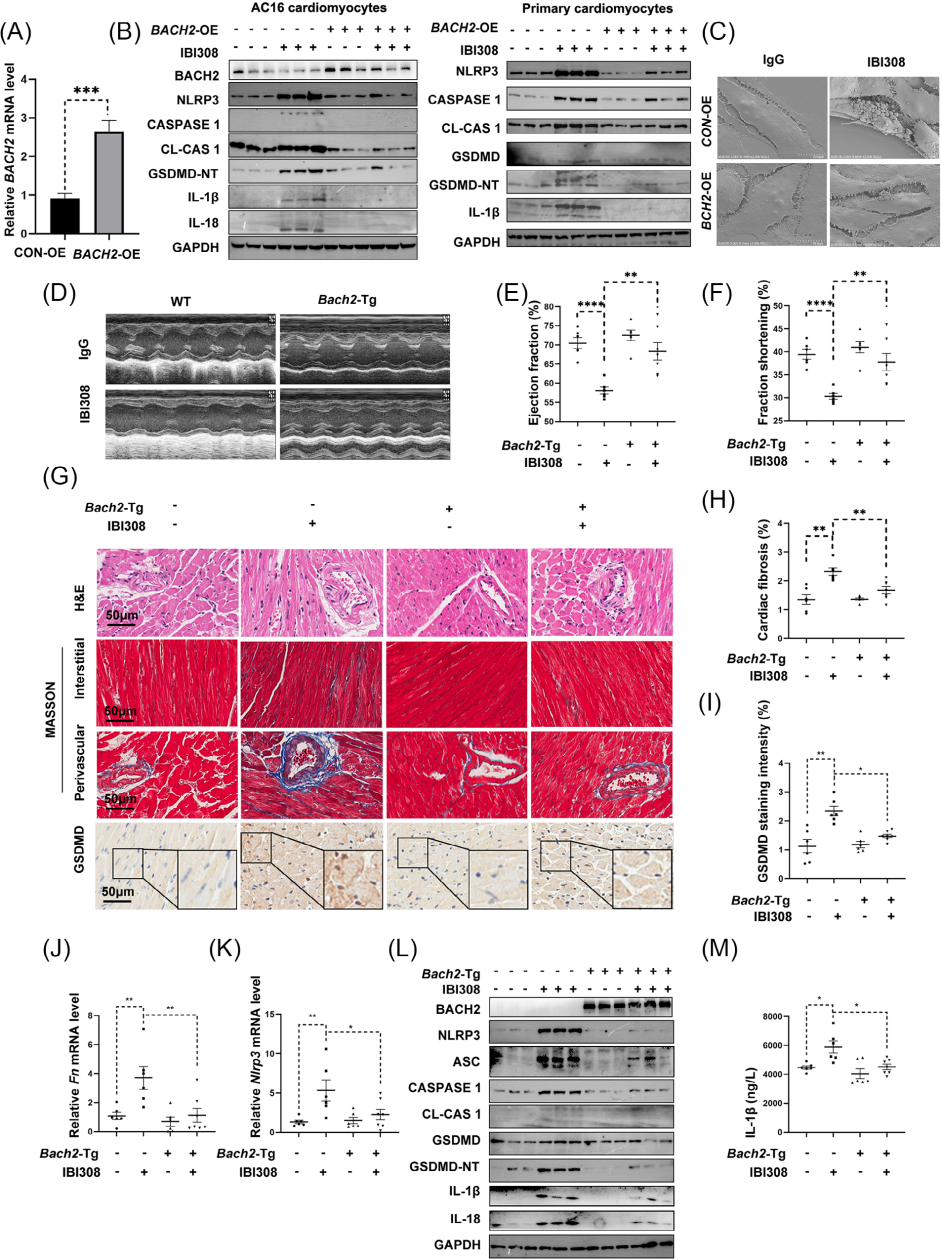
Overexpression of BACH2 alleviates IBI308‐induced myocyte pyroptosis and cardiotoxicity. (A) Relative mRNA levels of *BACH2* in AC16 myocytes stably overexpressing BACH2 (BACH2‐OE). CON‐OE indicates cells overexpressing the control plasmid vector. (B) Western blot analysis of the NLRP3 inflammasome and pyroptosis in *BACH2*‐OE cells and *CON*‐OE cells with or without IBI308 treatment (4 µg/µL) for 24 h. (C) Representative scanning electron microscope (SEM) images of AC16 myocytes with indicated treatments. (D–M) *Bach2* cardiac‐specific transgenic (*Bach2*‐Tg) mice were established and treated with IBI308 (50 mg/kg, weekly for a month) (*n* = 6–7 per group). (D) Representative M‐mode echocardiographic images of *Bach2*‐Tg mice and WT mice. (E) and (F) Ejection fraction and fraction of shortening measured in each group. (G) HE staining, Masson Trichrome staining and immunohistochemistry staining of GSDMD from each mouse. Scale bar = 50 µm. (H) Quantification of Masson Trichrome staining in 2G. (I) Quantification of GSDMD staining intensity in 2G. (J) Relative mRNA levels of *Fn*. (K) Relative mRNA levels of *Nlrp3* in the mouse hearts. (L) Western blot analysis of the BACH2 and pyroptosis in the mouse hearts. (M) ELISA analysis of IL‐1β in the mouse hearts. *p < .05; **p < .01; ***p < .001; ****p < .0001 as indicated.

BACH2 was downregulated by IBI308; we then tried to recover BACH2 expression to verify BACH2 function in ICIs‐induced cardiotoxicity. To this end, we established AC16 cardiomyocytes and primary cardiomyocytes stably overexpressing *BACH2* (Figure 3A,B). It was detected that the IBI308‐induced upregulation of NLRP3, ASC, CASPASE 1, and GSDMD proteins was all blunted by *BACH2* overexpression (Figure 3B). Scanning electron microscopy revealed that there were fewer bubble‐like protrusions in the *BACH2*‐overexpressing myocytes (Figure 3C). The anti‐pyroptotic effect of BACH2 was also demonstrated in vivo when we constructed *Bach2*‐Tg mice by crossing *Myh6*‐Cre mice with *Rosa26^LSL/LSL^* mice. Cardiac‐specific *Bach2* overexpression did not cause remarkable effects on the cardiac pathophysiology. However, while exposed to IBI308, the *Bach2*‐Tg mice significantly rescued IBI308‐induced decreases of EF and FS values (Figure 3D–F), mitigated cardiac fibrosis and pyroptosis (Figure 3G–L) as compared with WT mice. Moreover, IBI308‐elicited increases of IL‐1β release were significantly rescued in the B*ach2*‐Tg mouse hearts (Figure 3M). In support of these findings, we also upregulated cardiac *Bach2* expression using an adeno‐associated virus 9 (AAV9)‐delivered *Bach2* overexpression approach and found that it significantly improved cardiac function, attenuated cardiac fibrosis, and suppressed pyroptosis caused by IBI308 treatment (Figure S5).

BACH2 is well documented to reside in immune cells and regulate immune responses.^36^ To investigate whether BACH2‐regulated ICIs cardiotoxicity depends on immune regulation or not, we overexpressed *Bach2* in nude mice with congenital immune defects. It was observed that IBI308 still induced cardiac injuries and pyroptosis in these nude mice, reinforcing the notion that ICIs may directly pose cardiotoxicity through mechanisms independent of the adaptive immune regulation. More importantly, compared with the nude mice without *Bach2* overexpression, the *Bach2*‐OE nude mice showed improved cardiac function (Figure S6A–C) and reduced cardiac fibrosis (Figure S6D,E) and pyroptosis (Figure S6D,F–H) induced by IBI308 treatment. These findings suggested that the protection of BACH2 against ICIs‐induced cardiotoxicity was adaptive immune‐independent. Furthermore, AAV9‐*Bach2* failed to produce beneficial effects in the *Nlrp3*‐KO mice (Figure S7), indicating that NLRP3 signalling lies downstream of BACH2.

**FIGURE 4:**
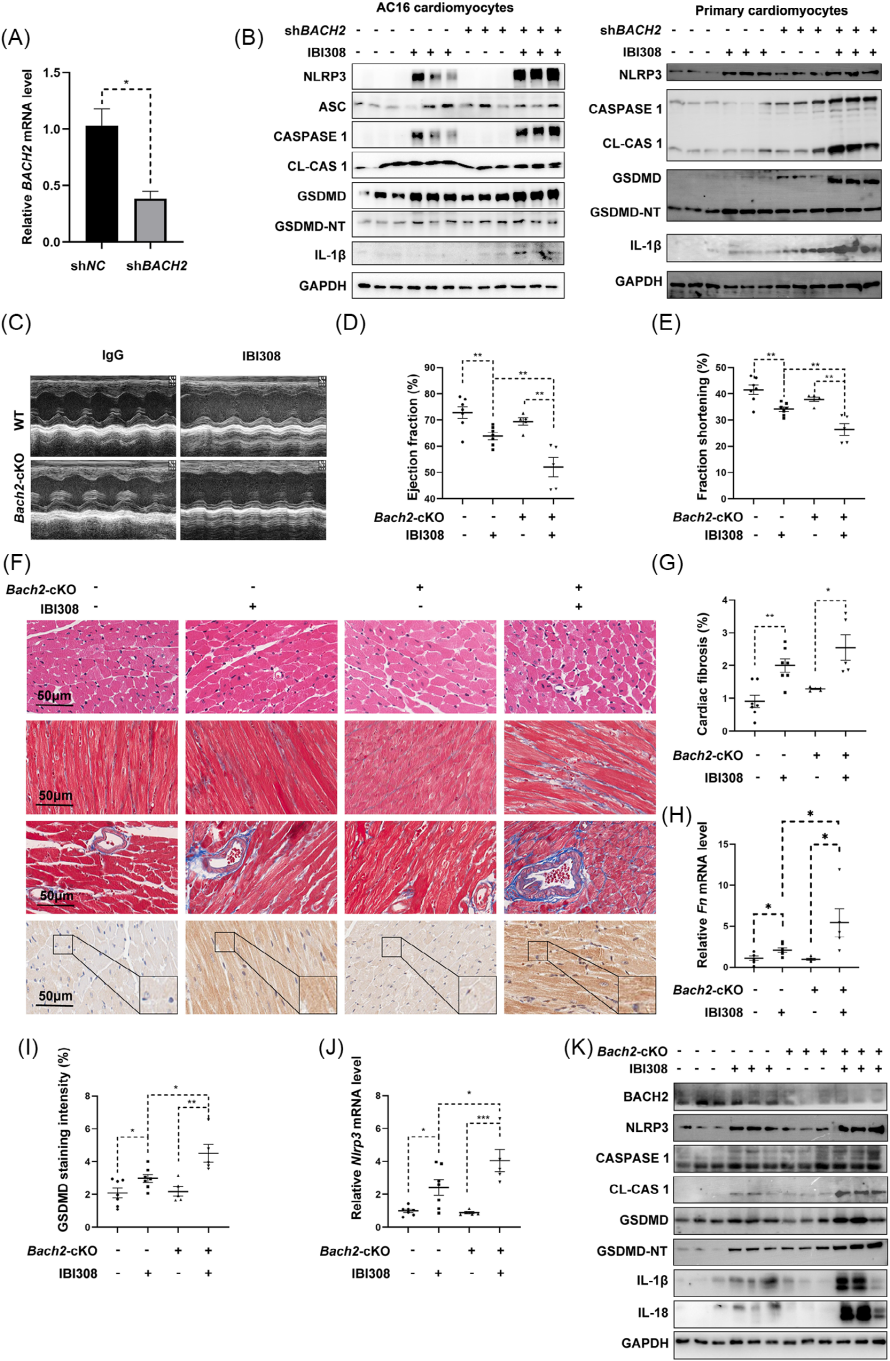
Cardiac conditional knockout of BACH2 (*Bach2‐cKO*) aggravates IBI308‐induced cardiomyocyte pyroptosis and cardiotoxicity. (A) Relative mRNA levels of *BACH2* in AC16 myocytes after stable silence of BACH2 (sh*BACH2*). sh*NC* indicated cells with a negative control shRNA. (B) Western blot analysis of the NLRP3 inflammasome and pyroptosis in sh*BACH2* cells and sh*NC* cells with or without IBI308 treatment (4 µg/µL) for 24 hours. (C–K), *Bach2*‐ cKO mice were established and treated with IBI308 (50 mg/kg, weekly for a month) (*n* = 5–7 per group). (C) Representative M‐mode echocardiographic images of *Bach2*‐cKO mice and WT mice. (D) and (E) Ejection fraction and fraction of shortening of each group. (F) HE staining, Masson Trichrome staining and immunohistochemistry staining of GSDMD from each mouse. Scale bar = 50 µm. (G) Quantification of cardiac fibrosis. (H) Relative mRNA levels of *Fn* in the mouse hearts. (I) Quantification of GSDMD staining intensity. (J) Relative mRNA levels of *Nlrp3* in the mouse hearts. (K) Western blot analysis of BACH2 and pyroptosis proteins in the mouse hearts. *p < .05; **p < .01; ***p < .001 as indicated.

On the other hand, silencing of *BACH2* by a specific shRNA (Figure 4A) aggravated IBI308‐induced pyroptosis in AC16 cells and primary cardiomyocytes (Figure 4B). We then constructed cardiomyocyte‐specific *Bach2* knockout (*Bach2*‐cKO) mice by breeding *Bach2^flox/flox^* mice with *Myh6*‐Cre mice. Compared with *flox/ flox* mice, the *Bach2*‐cKO mice developed more severe cardiac function (Figure 4C–E), cardiac fibrosis (Figure 4F–H), and cell pyroptosis (Figure 4F,I–K) following IBI308 treatments. Collectively, these data indicated that cardiac BACH2 could sufficiently protect against ICIs‐induced cardiotoxicity both in vitro and in vivo.

### 2.7 BACH2 protects against ICIs cardiotoxicity by promoting *GRSF1* transcription

**FIGURE 5:**
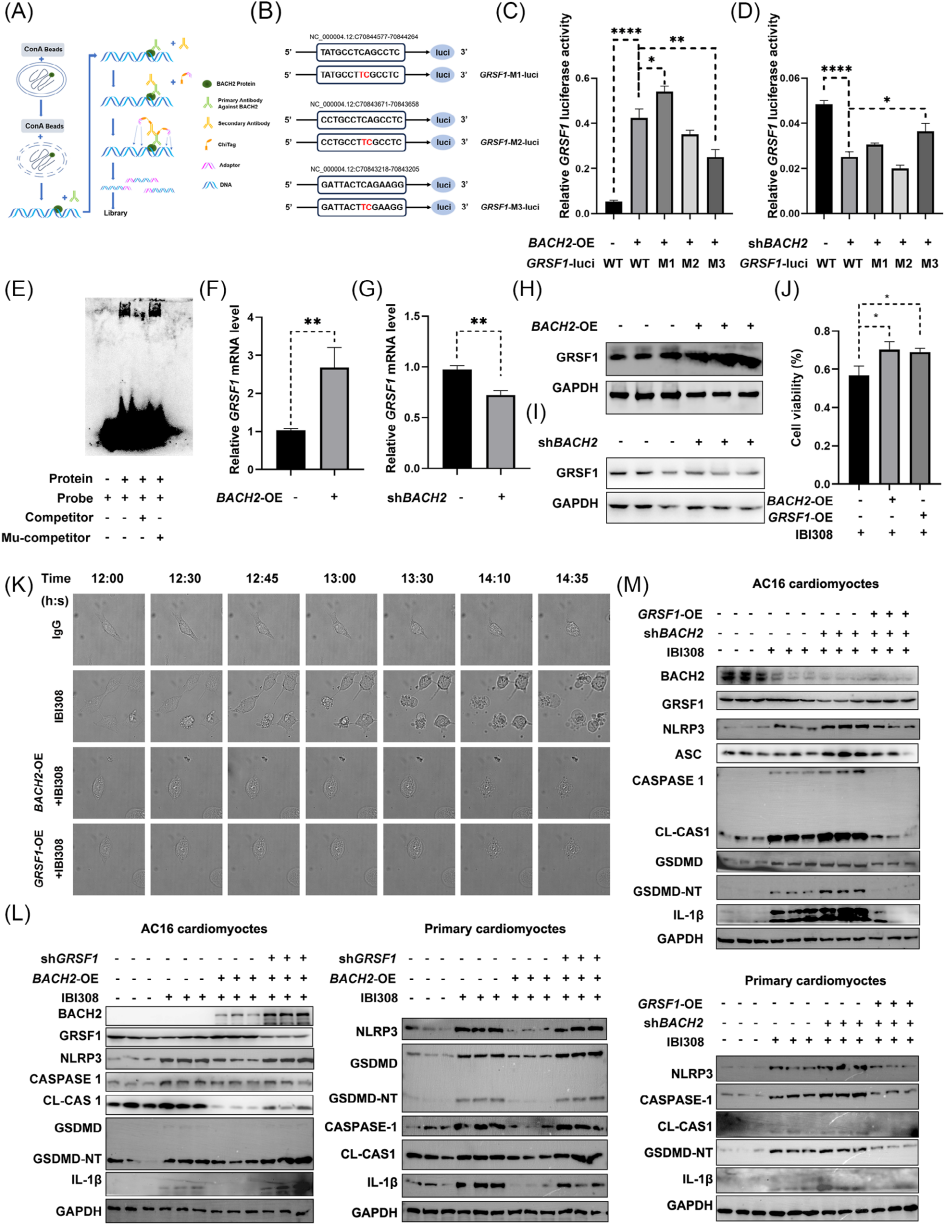
BACH2 exerts protective effects by directly enhancing the transcription of *GRSF1*. (A) Schematic diagram of CUT&TAG experiments. (B) Schematic illustration of putative binding sites of BACH2 in the promoter of *GRSF1* (*GRSF1*‐WT‐luci) and the site mutations (*GRSF1*‐M1‐luci, *GRSF1*‐M2‐luci, *GRSF1*‐M3‐luci). Mutated sites are highlighted in red. (C) and (D) Effects of overexpression or silence of *BACH2* on *GRSF1* luciferase activities when the three binding sites of BACH2 were mutated separately (denoted as M1, M2 and M3). (E) Electrophoretic mobility shift assay (EMSA) showing BACH2 binding to the third site of *GRSF1* promoter. (F) and (G) Relative mRNA levels of *GRSF1* in AC16 cells with overexpression or silence of *BACH2*. (H) and (I) Western blot analysis of GRSF1 in AC16 myocytes with overexpression or silence of *BACH2*. (J) Cell viability of AC16 cardiomyocytes with overexpression of *BACH2* or *GRSF1* under the treatment of IBI308 (4 µg/µL) for 24 h. (K) Time‐lapse microscopy of IBI308‐treated (4 µg/µL) AC16 myocytes with indicated treatments. Time duration = h: min. (L) and (M) Western blot analysis of pyroptosis proteins in AC16 cardiomyocytes and primary cardiomyocytes when *BACH2* and *GRSF1* were modulated as indicated, in the presence or absence of IBI308 (4 µg/µL) for 24 h. *p < .05; **p < .01; ***p < .001; ****p < .0001 as indicated.

Next, we tried to elucidate the mechanisms underlying BACH2 biological functions. Given that BACH2 is a transcription factor, we asked whether it directly bound to the promoter regions of *NLRP3*, *ASC*, *CASPASE 1*, or *GSDMD*. However, no binding sites were detected (data not shown). We then performed a cleavage under target and tagmentation (CUT&Tag) experiment and next‐generation sequencing (Figure 5A). Among these results, G‐Rich RNA sequence binding factor 1 (*GRSF1*), a gene involved in inflammation and regulation of nuclear factor κB (NF‐κB),^41^ ranked top. After searching the JASPAR database, we identified three putative binding sites of BACH2 in the *GRSF1* promoter region (Figure 5B). Dual‐luciferase assays confirmed that overexpression of BACH2 promoted, whereas silencing of BACH2 reduced *GRSF1* transcription. Mutation of the third binding site (GRSF1‐M3), but not the other two, abolished the regulatory effects of *GRSF1* by BACH2 (Figure 5B–D). Electrophoretic mobility shift assays (EMSA) further confirmed the direct binding of BACH2 to the third site of the *GRSF1* promoter (Figure 5E). Expression of GRSF1 was positively regulated by BACH2 at both mRNA (Figure 5F,G) and protein levels (Figure 5H,I).

Cell viability assessment demonstrated that overexpression of *BACH2* or *GRSF1* rescued the IBI308‐induced decline in cell viability (Figure 5J). Time‐lapse microscopy showed that IBI308‐treated myocytes began to detach approximately 12.5 h after drug exposure, and displayed multiple bubble‐like protrusions and progressed to pyroptosis approximately 13.5 h after drug exposure. Overexpression of either *BACH2* or *GRSF1* consistently inhibited IBI308‐induced cell morphology changes (Figure 5K). More importantly, the protective effect of *BACH2* overexpression on IBI308‐triggered pyroptosis was blunted by genetic silencing of *GRSF1* (Figure 5L), while the effect of *BACH2* depletion on IBI308‐triggered pyroptosis was completely reversed by *GRSF1* overexpression (Figure 5M).

**FIGURE 6:**
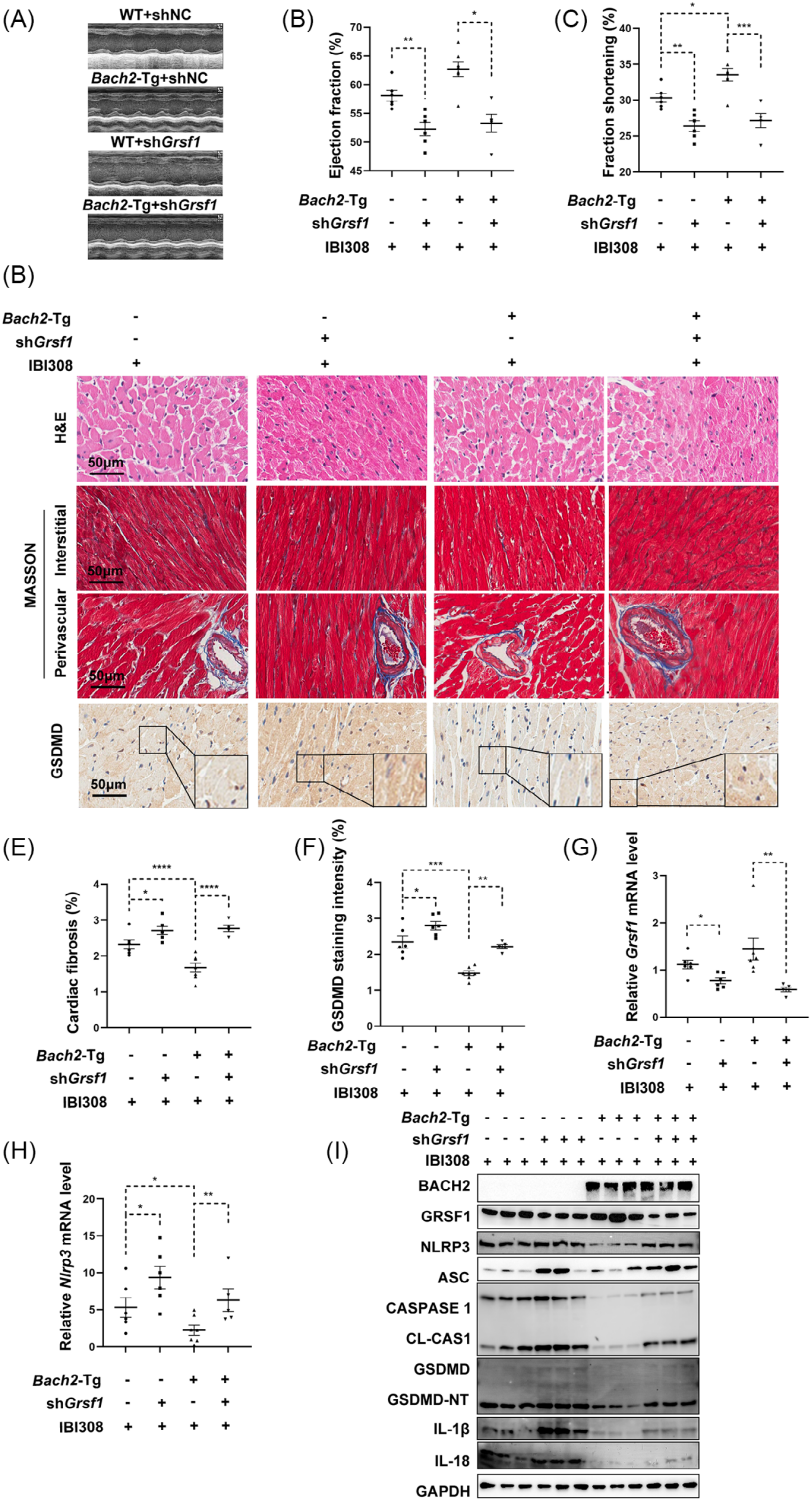
Silence of *Grsf1* dampens the beneficial role of BACH2 in IBI308‐induced pyroptosis and cardiotoxicity. *Bach2*‐Tg mice and WT mice were injected with AAV‐sh*Grsf1* or negative control virus through the tail vein, followed by treatment of IgG or IBI308 (50 mg/kg, weekly for a month) (*n* = 5–7 per group). (A) M‐mode echocardiography was conducted, and representative images were presented. (B) and (C) Ejection fraction and fraction of shortening of each mouse. (D) HE staining, Masson Trichrome staining and immunohistochemistry staining of GSDMD from each mouse. Scale bar = 50 µm. (E) Quantification of cardiac fibrosis. (F) Quantification of GSDMD staining intensity. (G) and (H) Relative mRNA levels of *Grsf1* and *Nlrp3*. (I) Western blot analysis of BACH2, GRSF1, and pyroptosis proteins in the hearts of mice. *p < .05; **p < .01; ***p < .001; ****p < .0001 as indicated.

We then injected AAV9‐*αMhc‐shGrsf1* into *Bach2*‐Tg mice and its counterpart WT mice. It was shown that cardiac depletion of *Grsf1* significantly blunted *Bach2* overexpression‐mediated protective effects, as measured by the improved heart function (Figure 6A–C), cardiac fibrosis (Figure 6D,E), and pyroptosis (Figure 6D,F–I).

GRSF1 functions through the NF‐κB signalling.^41^ Since GRSF1 acts as an RNA‐binding protein, we searched the database and found that *NFκBIB*, *NFκBID*, and *NFκBIE* might be the downstream targets of GRSF1. Therefore, we further conducted RIP experiments and proved that GRSF1 only binds to *NFκBIB* (Figure S8A). To test whether BACH2/GRSF1 regulates pyroptosis via the NF‐κB signalling, a specific NF‐κB inhibitor, JSH23, was used to co‐treat myocytes. It was demonstrated that IBI308 induced NF‐κB phosphorylation. Overexpression of *BACH2* rescued the IBI308‐enhanced NF‐κB phosphorylation when *GRSF1* was present (Figure S8B). Similarly, while depletion of *BACH2* deteriorated IBI308‐induced NF‐κB phosphorylation, overexpression of *GRSF1* reversed this effect (Figure S8C). Importantly, the pyroptosis caused by IBI308 treatment was exacerbated after *GRSF1* depletion, but was alleviated by JSH23 co‐administration (Figure S8D). These data together suggested that BACH2/GRSF1 suppresses ICIs‐induced myocyte pyroptosis through regulating the NF‐κB signalling.

### 2.8 Lipoic acid attenuates ICIs‐induced pyroptosis and cardiotoxicity by upregulating BACH2 expression

**FIGURE 7:**
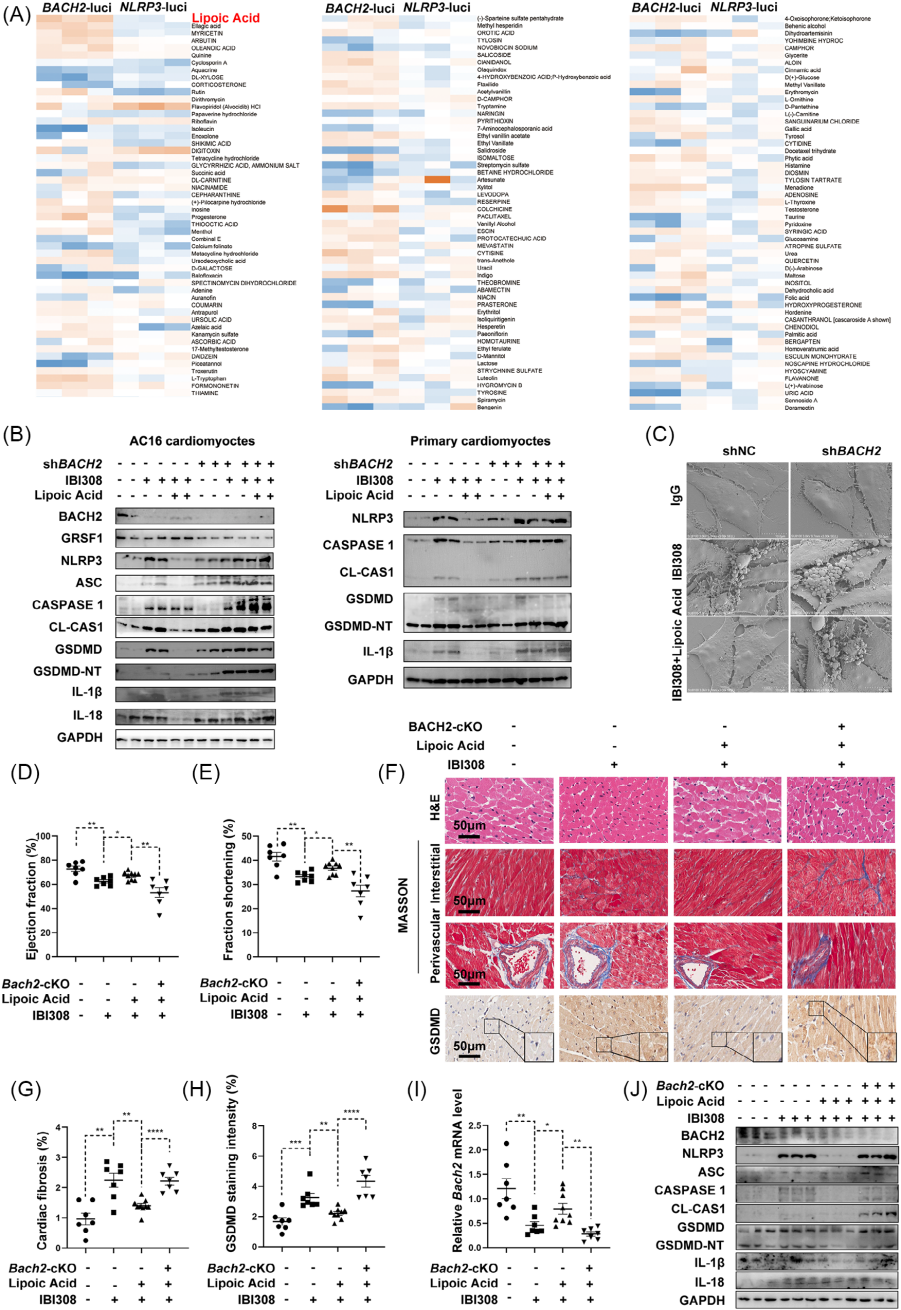
Lipoic acid may serve as a specific BACH2 transcriptional inducer that attenuates ICIs‐induced pyroptosis and cardiotoxicity. (A) Heat map of the relative *BACH2* and *NLRP3* luciferase activities when natural products (1 µM) were added to AC16 cells treated with IBI308 (4 µg/µL) for 24 h. (B) Western blot analysis of BACH2, GRSF1 and pyroptosis proteins in AC16 cells and primary cardiomyocytes with indicated treatments. IBI308 = 4 µg/µL for 24 h. Lipoic acid = 10 µM for 24 h. (C) Representative scanning electron microscope images of human AC16 myocytes with indicated treatments. (D–J) Lipoic acid (4 mg/kg, daily for 5 weeks) was co‐administered with IBI308 (50 mg/kg, weekly for a month) to inject into *Bach2*‐cKO mice and WT mice (*n* = 7–9 per group). (D) and (E) Ejection fraction and fraction of shortening of each mouse were measured in M‐mode echocardiography. (F) HE staining, Masson Trichrome staining and immunohistochemistry staining of GSDMD from each mouse. Scale bar = 50 µm. (G) Quantification of Masson Trichrome staining in 7F. (H) Quantification of GSDMD staining intensity in 7F. (I) Relative mRNA levels of *Bach2*. (J) Western blot analysis of BACH2 and pyroptosis proteins in mouse hearts. *p < .05; **p < .01; ***p < .001; ****p < .0001 as indicated.

Next, luciferase reporter assays driven by the promoter of *BACH2* and *NLRP3* were included in 160 natural products from the No.52 small‐molecule drug library (Chinese Academy of Science, Shanghai, China) to identify potential BACH2 activators that could prevent IBI308‐induced myocardial pyroptosis and cardiotoxicity. Among these drugs, lipoic acid, ellagic acid and myricetin significantly upregulated *BACH2* transcription and downregulated *NLRP3* transcription simultaneously. Lipoic acid was the most potent one (Figure 7A).

No significant changes in cell viability after treatment with lipoic acid were observed, indicating no myocardial toxicity existed (Figure S9A). RT‐qPCR confirmed that lipoic acid downregulated *NLRP3* (Figure S9B) and upregulated *BACH2* (Figure S9C). WB analyses demonstrated that lipoic acid attenuated IBI308‐induced pyroptosis (Figure S9D). A previous study has identified ALDH2 as a target of lipoic acid.^42^ We thus knocked down *ALDH2* in AC16 myocytes and found that the protective effect of lipoic acid against IBI308‐induced pyroptosis was retained (Figure S9E), hinting that other targets existed besides ALDH2. As shown in Figure 7B,C, IBI308‐induced pyroptosis could be inhibited by lipoic acid treatment in WT cardiomyocytes, but this protective effect was blocked by *BACH2* deficiency, further indicating BACH2 as its potential target. In vivo studies suggested that lipoic acid reversed IBI308‐elicited cardiac function impairment (Figure S9F; Figure 7D,E), cardiac fibrosis (Figure 7F,G), and pyroptosis (Figure 7F,H–J). However, lipoic acid‐mediated protective effects were abolished in *Bach2*‐cKO mice (Figure 7D–J). Moreover, although the protective effect of lipoic acid was abrogated by depletion of *BACH2*, it was rescued after re‐expression of *GRSF1* (Figure S9G,H). These observations suggested that lipoic acid attenuates ICIs‐induced cardiotoxicity and pyroptosis by upregulating the BACH2/GRSF1 pathway.

## 3 DISCUSSION

**FIGURE 8:**
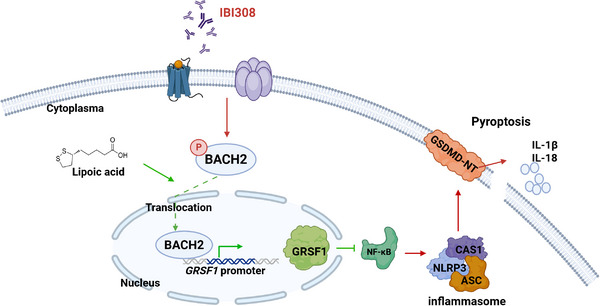
Schematic illustration of this study. In physiological conditions, BACH2 translocates to the nucleus to directly bind to the specific site on the promoter of *GRSF1* and inhibits NF‐κB‐NLRP3 signalling, whereas under ICIs (i.e., IBI308) treatments, the immunotherapy drug promotes the phosphorylation of BACH2, resulting in retention of BACH2 at cytoplasm. Small‐molecular compounds, such as lipoic acid, may specifically activate BACH2 and prevents from ICIs‐induced myocyte pyroptosis.

By gathering data from clinical patients, mouse models and cell culture, the present study revealed that myocyte pyroptosis predominantly caused a series of early cardiotoxic effects elicited by common ICIs. ICIs induced myocyte pyroptosis and toxicity independent of the presence of PD‐1 or the adaptive immune system. Cardiomyocyte‐specific BACH2 could potently protect against these cardiotoxic effects by directly enhancing GRSF1 transcription and regulating the downstream NF‐κB signalling. Lipoic acid could serve as a potential BACH2 transcriptional inducer that reversed IBI308‐induced pyroptosis and cardiotoxicity (Figure 8).

In our study, patients treated with ICIs exhibited elevated cTNT and IL‐18, suggesting subclinical myocardial stress. The absence of NT‐proBNP elevation further indicated that myocardial stress was in the early stage. The discrepancy between IL‐18 and IL‐1β may be attributed to the relatively low specificity of IL‐1β. It is widely involved in systemic inflammatory and infectious diseases, and the changes may be masked by interfering factors. Although there were no statistical differences in baseline characteristics, chemotherapies, and angiogenesis inhibitors between the two groups, some patients in the control group received ALK/ROS‐targeted drugs. ALK/ROS inhibitors increase cardiotoxicity, which is often manifested as arrhythmias and heart failure,^43^ especially in patients with cardiac comorbidity.^44^ NSCLC with driver mutations (such as EGFR, ALK, and ROS) has a lower degree of inflammation in the tumour microenvironment, while its relationship with peripheral inflammatory parameters has not been fully elucidated.^45^ The impact of tumour genetics and targeted drugs on biomarkers requires further exploration. There was also an imbalance in ECOG‐PS between the two groups. A higher PS score is frequently associated with more severe disease burden and systemic inflammation, influencing cardiac or inflammatory marker profiles. In our study, the PS scores of recruited patients ranged from 0 to 1, which, in mainstream clinical guidelines, are classified into the same group^46^, ^47^ and have a relatively limited impact on biomarkers. Experiments with a larger sample size are needed to eliminate the interference of the PS score.

In our study, the ICI drug alone could elicit side effects. This finding challenged previous studies using anti‐PD‐1 therapy in combination with radiation,^48^ or murine cardiac troponin I (cTNI) peptide^49^ as the mouse model to mimic ICIs cardiotoxicity, where the direct effect of anti‐PD‐1 therapy alone on the mouse heart was masked. Several lines of explanation may be conceived. First, we employed various commonly used ICIs and selected IBI308, which showed the most significant changes, and administered it at a higher dose. Second, while it is less probable to observe severe myocarditis through morphological approaches in anti‐PD‐1 therapy treatment alone,^50^ we chose to reflect the cardiotoxic effects in ways of early molecular events. In this way, we detected most of the cardiac injury from a pyroptotic perspective (pyroptosis proteins), which lies preceding the development of the morphologically severe lesions and may only represent the initial mild injury stage.

Many mechanisms have been raised in attempts to dissect the ICIs treatment‐associated cardiotoxicity, with immune system disorder being the most claimed attributor. For example, autoimmune T cells recognizing cardiac myosin,^14^ B cells producing cardiac autoantibodies,^15^ and inflammatory macrophage infiltration^16^ have been shown to participate in ICIs‐induced cardiotoxicity. Our research indicated that the effects of IBI308 persisted in nude mice, which means it does not entirely rely on adaptive immunity. Considering the complexity of the tumour microenvironment and its critical factor in anti‐tumour immunity,^51^, ^52^ the roles of innate immune components, such as macrophages, NK cells, and complement, still require further investigation. Since the ICI drug could elicit pyroptosis in AC16 cardiomyocytes and primary cardiomyocytes, we hypothesize that there exist inherent mechanisms within cardiomyocytes. Moreover, ICIs elicited myocyte pyroptosis and cardiac injuries even after *PD‐1* knockout, which indicates a PD‐1‐independent mechanism. PD‐1/PD‐L1 axis is far more complex than the simplistic view that PD‐L1 in tumour cells directly binds to PD‐1 in cytotoxic T lymphocytes. Both PD‐1 and PD‐L1 exert intrinsic functions within the tumour, immune and stromal populations.^53^ Previous studies have revealed B7 family members that function through an unknown receptor.^54^, ^55^, ^56^ Anti‐PD‐L1 monoclonal antibody also activates PD‐L1^+^NK cells to control tumour growth independent of PD‐1.^57^ Further studies are needed to explore whether there exists a receptor to mediate signal transduction or off‐target effects. These insights challenge the traditional mechanism and require a brand‐new view of side effects induced by ICIs without necessarily relying on the immune system or PD‐1.

The immunoregulatory factor BACH2 has long been considered to reside in immune cells and regulate B cell development, T cell differentiation,^58^ and Treg cell function.^36^ This study found that BACH2 in cardiomyocytes could directly regulate ICI‐associated cardiac pathophysiology even in nude mice with adaptive immune defects. This is consistent with our previous findings that BACH2 in cardiomyocytes could transcriptionally repress *Rip1* and *Rip3* expression and protect against diabetic heart injuries,^39^ confirming that BACH2 is an endogenous heart guardian. As a transcription factor, nucleus‐location of BACH2 is crucial for its transcriptional activity. However, IBI308 induced BACH2 phosphorylation and cytoplasmic retention, and thus decreased nuclear BACH2 levels. These findings explain the initiation process of IBI308‐induced myocyte pyroptosis, and more research can be conducted to identify the involved kinases and phosphorylation residues on BACH2.

*AKAP6*,^38^ *RIP1*, and *RIP3* ^39^ are known downstream targets of BACH2 in cardiac hypertrophy and diabetic cardiomyopathy, respectively. In this study, *GRSF1* was identified as a novel target of BACH2, as evidenced by the finding that BACH2 could bind to *GATTACTCAGAAGG* sequences on the promoter of *GRSF1* and promote its transcription. GRSF1 belongs to the heterogeneous nuclear ribonucleoprotein F/H family,^59^ and is crucial for maintaining mitochondrial function. Absence of GRSF1 impaired mitochondrial respiration and increased reactive oxygen species (ROS) levels, causing DNA damage, mTOR and NF‐κB activation, inflammation, growth suppression, and senescence.^41^ We found that IBI308‐induced pyroptosis was exacerbated by *GRSF1* interference, which was alleviated by administration of the NF‐κB antagonist JSH23. RIP experiments proved that GRSF1 binds to *NFκBIB*. As an RNA‐binding protein, GRSF1 directly interacts with the G‐tracts of mRNAs to regulate their stability and function at the post‐transcriptional level,^60^ indicating that the NF‐κB pathway lies downstream of the BACH2/GRSF1/NFκBIB during ICIs‐induced pyroptosis.

Considering the endogenous cardioprotective roles of BACH2 in ICIs cardiotoxicity, we further identified lipoic acid, a small molecular compound, as a potential BACH2 transcriptional inducer that could directly upregulate BACH2 expression and inhibit IBI308‐induced myocyte pyroptosis and cardiotoxicity. Lipoic acid, chemically known as 1,2‐dithiolane‐3‐pentanoic acid, is synthesized in mitochondria^61^ and obtained through diet. It functions as a cofactor for the pyruvate dehydrogenase complex and α‐ketoglutarate dehydrogenase.^62^ Lipoic acid has beneficial effects in various cardiovascular diseases.^63^, ^64^, ^65^, ^66^ For example, lipoic acid protected against diabetic cardiomyopathy,^67^ ischemia–reperfusion injury,^68^ and pressure overload‐induced heart failure^42^ by restoring ALDH2 activity. Lipoic acid also exhibits multifunctional anti‐tumour activity in several cancer models, regulating carcinogens, invasion, migration, EMT and cancer stemness, as well as enhancing the efficacy and reducing the side effects of tumour treatment.^69^ In immune therapy, lipoic acid induces nuclear PD‐L1 translocation, thus suppressing tumorigenesis, boosting anti‐tumour immunity, and overcoming resistance to ICIs.^70^ Our study showed that lipoic acid attenuated pyroptosis caused by ICIs. The beneficial effects of lipoic acid were dismissed when *BACH2* but not *ALDH2* was depleted in cardiomyocytes, further indicting BACH2 was a target of lipoic acid. Indirect functions via the Nrf2 pathway^71^ or by reducing ROS^72^ cannot be ruled out. Thus, our study may provide evidence for extending lipoic acid into clinical application in Cardio‐Oncology. With the development of proteomics,^73^ network pharmacology prediction,^74^ molecular docking and molecular dynamics simulation techniques,^75^ more personalized drugs may emerge in the future.

## 4 CONCLUSION

ICIs treatment induced myocyte pyroptosis and cardiac dysfunction, independent of PD‐1 or adaptive immune. BACH2 protected against the ICIs‐induced myocyte pyroptosis and early cardiotoxicity by transcriptionally activating *GRSF1*, thus suppressing NF‐κB cascades. BACH2‐targeted small‐molecular transcriptional inducers, such as lipoic acid, might be a promising therapeutic agent used against ICIs‐induced cardiac side effects.

## 5 MATERIALS AND METHODS

### 5.1 Human subjects

This study adhered to the principles of the Declaration of Helsinki and was approved by the Ethical Review Board at Zhongshan Hospital, Fudan University (approval no.: B2021‐128). During the period of March 2021 to March 2022, a total of 269 patients with advanced NSCLC were admitted to the Department of Respiratory Medicine, Zhongshan Hospital. An aliquot of 200 µL serum samples before patient discharge was collected for analysis, and medical history and biochemical detection results were also collected from each patient. Patients with previous coronary heart disease (CAD), cardiomyopathy, valvular heart diseases, heart failure (HF), severe infection, and former irAEs (rash, hyperthyroidism, hypothyroidism, hepatotoxicity, diarrhea, colitis, nephritis, pneumonitis, myocarditis, arrhythmias, hematologic toxicity, myositis, vasculitis, and peripheral neuropathy) were excluded. Finally, 166 serum samples were included for analysis, of which 111 patients received ICIs treatments, and 55 patients did not. Among the patients treated with ICIs, 108 received anti‐PD‐1 drugs, 2 received anti‐PD‐L1 drugs, and 1 received an anti‐PD‐L1 drug combined with anti‐CTLA4 drug. For the purpose of this study, we summarized number of patients with elevated cardiac enzyme levels and transient electrocardiograph (ECG) abnormalities. Elevation of cardiac enzyme level was considered when the value of any myocardial enzymes (cTnT, CK‐MB, CK‐MM) exceeded the upper reference range. When patients were recorded of any non‐sinus rhythm, they were considered to have electrocardiographic (ECG) abnormalities.

### 5.2 Animal models and treatments

WT C57BL/6 mice were purchased from Shanghai Laboratory Animal Center (SLAC, Shanghai, China). The Rosa26 site‐directed knock‐in mice with conditional overexpression of CAG‐LSL‐Bach2‐m‐WPRE‐pA gene were constructed using CRISPR/Cas9 technology by Shanghai Model Organisms Center (*Rosa26^LSL / LSL^* mice). *Bach2^flox/flox^* mouse was a kind gift from Dr Chuanxin Huang at Shanghai Jiao Tong University. BALB/c‐nu mice were purchased from Gempharmatech Co., Ltd. Animal studies were conducted in compliance with the requirements of the Animal Care Committee at Zhongshan Hospital, Fudan University.

1. To study the cardiotoxicity of IBI308 and the role of pyroptosis in the cardiotoxicity induced by IBI308, 4‐week‐old male WT mice, Gsdmd‐KO mice and Nlrp3‐KO mice were intraperitoneally (i.p.) injected with 100 µL of IgG or IBI308 (50 mg/kg) weekly for a month.
2. To study the role of BACH2 in IBI308‐induced cardiotoxicity, Bach2 cardiac knock‐in mice (Bach2‐Tg) mice and Bach2 cardiac knockout mice (Bach2‐cKO) mice were utilized. The α‐myosin heavy chain (α‐MHC)‐MerCreMer transgenic mice (α‐MHC‐MCM, short for Cre) were crossed with the Rosa26LSL/LSL mice or Bach2flox/flox mice. Thereafter, the mice were i.p. injected with tamoxifen (Sigma‐Aldrich) at a dose of 75 mg/kg per day for five consecutive days to generate Bach2‐Tg mice or Bach2‐cKO mice. After 2 weeks of recovery, the mice were i.p. injected with 100 µL of IgG or IBI308 (50 mg/kg) weekly for a month.
3. In addition, adeno‐associated virus serotype 9 (AAV9)‐mediated cardiac BACH2 expression mice were utilized. AAV9‐α myosin heavy chain(αMhc)‐Bach2 and the AAV9‐Gfp control virus were purchased from HANBIO Inc. 4‐week‐old C57BL/6 mice were injected with AAV9‐αMhc‐Bach2 or control virus through the tail vein. Four weeks after AAV9 injection, mice were i.p. injected with IgG or IBI308 for 4 weeks as abovementioned.
4. To study the role of the immune mechanism in IBI308‐induced cardiotoxicity, BALB/c‐nu congenitally adaptive immune‐deficient mice aged 4 weeks old were injected with AAV9‐αMhc‐Bach2 or control virus through the tail vein, followed by i.p. injection of IgG or IBI308 for 4 weeks.
5. To study whether IBI308‐induced cardiotoxicity was dependent on PD‐1, Pd‐1‐KO mice and C57BL/6JCya‐Pdcd1em1/Cya (hPD‐1) mice purchased from Cyagen Biosciences Inc. were i.p. injected with 100 µL of IgG or IBI308 (50 mg/kg) weekly for a month.
6. To verify the role of GRSF1, Bach2‐Tg mice and Rosa26LSL/LSL mice were injected i.v. with AAV9‐αMhc‐shGrsf1 or AAV9‐shNC‐Gfp. Four weeks after AAV9 injection, mice were i.p. injected with IgG or IBI308 for 4 weeks as abovementioned.
7. To study the effects of lipoic acid, Bach2‐cKO mice and Bach2flox/flox mice were pretreated with vehicle or lipoic acid (4 mg/kg) daily, 1 week before IBI308 injection, followed by consecutive i.p. injection with vehicle or lipoic acid during the treatment of IBI308. Especially, on the day of IBI308 treatment, lipoic acid was pretreated 1 h before IBI308.

On the last day of treatment, blood and organs were collected.

### 5.3 Enzyme‐linked immunosorbent assay

Blood collected from patients and mice was centrifuged to separate serum, which was stored at −80°C until use. Commercial ELISA kits from Yu Bo Biotech Co., Ltd. were used to detect serum IL‐1β, IL‐18, LDH, TGFβ1, and CTGF levels according to the manufactural instruction.

### 5.4 Echocardiographic analysis

The Vevo2100 instrument (VisualSonics, Inc.) was used to detect the short‐axis of the left ventricle at a 400 bpm heart rate, and four consecutive cardiac cycles were reported. EF and FS were calculated by the Teichholz method.

### 5.5 Histological analysis

Hematoxylin and eosin (HE) staining and Masson trichrome staining were performed on the cross‐sectioned hearts. The images were captured by a Nikon microscope and analyzed using ImageJ software (National Institutes of Health).

### 5.6 Immunohistochemistry and immunofluorescence analysis

Immunohistochemistry and immunofluorescence were conducted as described in our previous research.^34^ The images were captured, and the positive staining areas were assessed using the ImageJ software.

### 5.7 Measurement of ferrous ion

The concentration of ferrous ions in heart tissues was detected with the ferrous ion content assay kit from Solarbio in accordance with the instructions.

### 5.8 Cell culture

Human AC16 myocytes were purchased from National Collection of Authenticated Cell Cultures (Cat no.: SCSP‐555), and primary neonatal mouse ventricular myocytes (NMVMs) were isolated from the newborn C57BL/6 mice (1–2 days old) as described before.^38^ Cells were primed with chemicals at indicated doses or for the indicated hours.

### 5.9 Construction of PD‐1 KO AC16 cells

CRISPR/Cas9‐mediated gene editing was used to establish the *PD‐1*‐KO cells. Briefly, four sgRNAs targeting PD‐1 were designed following the 5’‐NGG principle (sequences listed in Table S1) and ligated into the green fluorescence‐emitting backbone. The four sgRNA plasmids were co‐transfected into AC16 cells. Two days later, cells with green fluorescence were sorted out by flow cytometry (BD biotechnology) and seeded into plates, a single cell per well. After the mono‐clony was formed, the DNA was extracted and detected by RT‐qPCR analysis and DNA sequencing to confirm that the PD‐1 gene was completely deleted. Furthermore, RT‐qPCR and western blot were performed to confirm the knockout efficiency.

### 5.10 Cell viability

For cell viability assessment, AC16 cells were added with cell counting kit‐8 (CCK‐8) solution (YEASEN Biotechnology) for 2 h, and the absorbance was detected at a wavelength of 540 nm using a microplate reader (Biotek).

### 5.11 Lentivirus‐mediated gene expression and plasmid transfection

Lentivirus was constructed in HEK‐293T cells and infected with primary cardiomyocytes or AC16 cells. Briefly, expressing genes or shRNAs were cloned into the basic pLVX‐puro or pLKO.1‐puro plasmids, respectively. The transfer plasmids were then co‐transfected with packaging plasmid and envelope plasmid (1:1:1) into HEK‐293T cells. After 72 h of incubation, the supernatants were collected, filtered and added into primary cardiomyocytes or AC16 cells with 5 µg/mL polybrene. To establish gene‐stable expressing cell lines, puromycin (5 µg/mL) was added to AC16 cells 48 h after the transfection. The related gene expressions were confirmed by real‐time quantitative PCR (RT‐qPCR) and western blot analysis. For plasmid transfection, the Lipofectamine reagent (Invitrogen) was used in accordance with the manufacturer's guidance. The siRNA sequences are listed in Table S2.

### 5.12 RNA extraction and RT‐qPCR analysis

Total RNA was extracted and reverse transcribed. Quantitative PCR was conducted^39^ using primers listed in Table S3.

### 5.13 Protein extraction and Western blot analysis

Total proteins were extracted using RIPA solution. Nuclear and cytoplasmic proteins were separated with a commercial kit purchased from Beyotime. Briefly, the cell membrane was destroyed by the plasma protein extraction reagent, and the supernatant was collected by a high‐speed centrifuge. Afterwards, the nuclear protein extraction reagent was added, and the nuclear proteins were extracted by vortexing at maximum speed. Western blot analyses were conducted using antibodies listed in Table S4 as previously described.^39^

### 5.14 Immunoprecipitation assay

To detect the phosphorylation of BACH2, proteins were extracted and incubated with normal IgG and a small quantity of Agarose G Beads (Beyotime) to avoid non‐specificity. After a transient and soft centrifuge, the supernatants were mixed with BACH2 primary antibodies overnight, after which Agarose G Beads were added for an additional 3 h. The precipitates were detected with SDS‐PAGE gel followed by immunoblotting.

### 5.15 Scanning electron microscopy

For SEM, the AC16 myocytes receiving indicated treatments were fixed in 2.5% glutaraldehyde, dehydrated through gradient ethanol and dried by the tertiary butanol method. Specimens were then sputter‐coated with gold‐palladium and imaged under a SU8010 microscope (Hitachi).

### 5.16 Cleavage under target and tagmentation

Commercial CUT&Tag kit (Novoprotein Scientific Inc.) was involved in this study. Briefly, about 1 × 10^5^ AC16 cells were collected and combined with concanavalin A (ConA) beads. After being permeabilized by digitonin, the ConA bead‐cell complexes were incubated with primary antibody, secondary antibody and the Tn5 transposase successively. After fragmentation, DNA was extracted for sequencing.

### 5.17 Site‐directed mutagenesis and dual luciferase reporter gene assay

The human *GRSF1* promoter was synthesized by Qingke Biotech and cloned into the pGL3.0 luciferase vector. Point mutations were then introduced using the Fast Mutagenesis Kit (TransGene) and confirmed by DNA sequencing. WT or the mutated plasmids, BACH2‐expressing or control plasmids, together with Rellina plasmids, were co‐transfected into HEK 293T cells. Firefly and rellina luciferase activities were detected (YEASEN), the absorbance of which was collected by BioTek hybrid reader.

### 5.18 Electrophoretic mobility shift assay

EMSA assays were conducted using Chemiluminescent EMSA Kits (Beyotime). Probes targeting the BACH2 binding sites on the GRSF1 promoter (forward 5′‐3′: CCTGATTACTCAGAAGGTCA, reverse 5′‐3′: GGACTAATGAGTCTTCCAGT, mutant forward 5′‐3′: CCTGATTACTTCGAAGGTCA, mutant reverse 5′‐3′: GGACTAATGAAGCTTCCAGT) were synthesized and labelled with biotin. Purified human BACH2 protein (AA618‐770) was incubated with biotin‐labelled probes. A competition assay was conducted with either an unlabelled biotin probe containing the BACH2 binding site or an unlabelled probe with a mutated BACH2 binding site.

### 5.19 Chromatin immunoprecipitation assay

Chromatin immunoprecipitation (ChIP) assays were performed with SimpleChIP Enzymatic Chromatin IP kits (Cell Signal Technology). Briefly, AC16 cells with different treatments were fixed with 1% formaldehyde to cross‐link proteins to DNA. Micrococcal nuclease and an ultrasonic homogenizer were used to digest chromatin and break the nuclear membrane. An antibody specific to BACH2 was added to co‐precipitate DNA‐protein complexes, which were further captured by protein G agarose. After reversal of cross‐links, DNAs were purified and quantified by PCR assays. The JASPAR database was searched to predict the BACH2 binding site on promoters of potential genes. The primers used in ChIP assays are listed in Table S5.

### 5.20 Small‐molecule drugs library screening

A total of 160 compounds from the No.52 small molecule drug library were purchased randomly from the Chinese Academy of Sciences. Human *BACH2* and *NLRP3* promoters were cloned into the pGL3.0 luciferase vector by Qingke Biotech, which were then co‐transfected with Renilla control plasmid into HEK 293T cells. After 48 h, 1 µM small‐molecule drugs were added and incubated for an additional 24 h. The transcriptional activities of *BACH2* or *NLRP3* were then detected with dual‐luciferase reporter assays.

### 5.21 RNA immunoprecipitation assay

RNA immunoprecipitation (RIP) assay was performed with a pure binding RNA immunoprecipitation kit (Geneseed Biotech. Co., Ltd.). Briefly, cells transfected with pCDNA3.1‐3×Myc‐GRSF1 plasmid (Miaoling Biotech) were lysed. After centrifugation, the supernatant was incubated with Protein A+G magnetic beads pre‐conjugated with primary antibody against Myc‐Tag (2276, Cell Signal Technology) or IgG overnight at 4°C with gentle rotation. The beads were washed, and the bound RNA was extracted. We searched RBPs databases (including HuRPA, RBP2GO, RBPDB, ATtRACT, hRBPome, RBPbase, and starBase) for RNAs that potentially bind with GRSF1. Purified RNA was reverse‐transcribed into cDNA and tested with RT‐qPCR analysis.

### 5.22 Statistics

The results were presented as mean ± standard errors (SEM). For numerical variables with homogenous variance and normal distribution, Student's *t*‐test and one‐way ANOVA, along with Bonferroni post hoc test, were applied; otherwise, Mann–Whitney test or Kruskal–Wallis test, followed by Dunn post hoc test, were used. For categorical variables, the chi‐squared or Fisher's exact test was applied where appropriate. Statistical analyses were performed using SPSS version 20.0 (IBMCorp) and GraphPad Prism version 8.0 (GraphPad). A *p*‐value < .05 was considered statistically significant.

## AUTHOR CONTRIBUTIONS

Pan Gao and Yunzeng Zou designed the project and provided funding. Mengying Cao performed most of the in vivo and in vitro experiments. Zilong Liu collected the human serum, and Di Zhao performed part of the in vivo mouse models. Hongyuan Zhang, Xinjie He, Wenhao Wang, and Xiaolin Wang helped perform the experiments. Pan Gao and Yunzeng Zou drafted and revised the manuscript. All authors revised and approved the manuscript.

## CONFLICT OF INTEREST STATEMENT

All authors declare no conflict of interest.

## ETHICS STATEMENT

The human serum study adhered to the principles of the Declaration of Helsinki and was approved by the Ethical Review Board at Zhongshan Hospital, Fudan University (approval no.: B2021‐128). The protocol for the animal study was approved by the Animal Care and Use Committee of Zhongshan Hospital, Fudan University (Approval Numbers: 2021–061 and 2023–154).

## Supporting information



Supporting Information

## Data Availability

All data are available from the corresponding authors upon reasonable request.
