# Supplementary material for: BACH2 alleviates immune checkpoint inhibitors‐induced cardiac pyroptosis via transcriptionally promoting GRSF1
Source: Clin Transl Med. 2026 Feb 10;16(2):e70618. doi: 10.1002/ctm2.70618 (PMC12889571; doi:10.1002/ctm2.70618)
Supplement: Supplementary file 1 — Supporting Information [file CTM2-16-e70618-s001.docx]

**Supplementary Materials**

**BACH2 alleviates immune checkpoint inhibitors-induced cardiac pyroptosis via transcriptionally promoting GRSF1**

**Running Title:** BACH2/GRSF1 alleviates ICIs-induced cardiotoxicity

**This file includes:**

Supplemental Table 1-5

Supplemental Figure 1-9

**Supplemental Materials**

**Supplemental Tables**

**Table S1. sgRNAs sequences**

| Gene name | Target sequence (5’-3’) |
| --- | --- |
| *PD-1 #1* | CTGCAGCTTCTCCAACACAT |
| *PD-1 #2* | CTTCCACATGAGCGTGGTCA |
| *PD-1 #3* | CAGTTCCAAACCCTGGTGGT |
| *PD-1 #4* | CTGAGGATGGACACTGCTCT |

**Table S2. siRNA sequences**

| Gene name | Target sequence (5’-3’) |
| --- | --- |
| *hBACH2* | CCTGTAGATCAAATCACAGAT |
| *hGRSF1* | GCCCAAGACATTATAAACTTT |
| *hALDH2* | GAGGACATCTATGATGAGTTT |

**Table S3. Primers used in qRT-PCR**

| Genes | Forward (5’-3’) | Reverse (5’-3’) |
| --- | --- | --- |
| *hBACH2* | AATACCAGCTTGCATGTACCAA | TTATCTTCCCGGAATGTGCTTG |
| *hPDL1* | TTATTATGCCTTGGTGTAG | ATCCATCATTCTCCCTTT |
| *hGRSF1* | GCCAGCGGTATGTGGAAGTAT | AGGCGAAGATTTGACCTGCAA |
| *hNLRP3* | GATCTTCGCTGCGATCAACAG | CGTGCATTATCTGAACCCCAC |
| *hASC* | TGGATGCTCTGTACGGGAAG | CCAGGCTGGTGTGAAACTGAA |
| *hCASP1* | ACACGTCTTGCCCTCATTATCT | ATAACCTTGGGCTTGTCTTTCA |
| *hGSDMD* | GAGTGTGGCCTAGAGCTGG | GGCTCAGTCCTGATAGCAGTG |
| *hIL-1β* | CCCTGCAGCTGGAGAGTGTGG | TGTGCTCTGCTTGAGAGGTGCT |
| *hIL-18* | ACAACCGCAGTAATACGGAGCA | TGTGCTCTGCTTGAGAGGTGCT |
| *hGAPDH* | CTGGGCTACACTGAGCACC | AAGTGGTCGTTGAGGGCAATG |
| *mBach2* | TCAATGACCAACGGAAGAAGG | GTGCTTGCCAGAAGTATTCACT |
| *mGrsf1* | CTGCCTGAGTACGAGTTGAGC | CCCTCCGTTTCCCATCTCTATTT |
| *mNlrp3* | ATTACCCGCCCGAGAAAGG | TCGCAGCAAAGATCCACACAG |
| *mAsc* | CTTGTCAGGGGATGAACTCAAAA | GCCATACGACTCCAGATAGTAGC |
| *mCasp1* | ACAAGGCACGGGACCTATG | TCCCAGTCAGTCCTGGAAATG |
| *mGsdmd* | CCATCGGCCTTTGAGAAAGTG | ACACATGAATAACGGGGTTTCC |
| *mIl-1β* | GCAACTGTTCCTGAACTCAACT | ATCTTTTGGGGTCCGTCAACT |
| *mIl-18* | GACTCTTGCGTCAACTTCAAGG | CAGGCTGTCTTTTGTCAACGA |
| *mGapdh* | AGGTCGGTGTGAACGGATTTG | TGTAGACCATGTAGTTGAGGTCA |
| *mTgfβ1* | TAAAGAGGTCACCCGCGTGCTAAT | ACTGCTTCCCGAATGTCTGACGTA |
| *mFn* | ACGGTTTCCCATTACGCCAT | TCATCCGCTGGCCATTTTCT |

**Table S4. Antibody information**

| Antibody | Company | Catalog No. |
| --- | --- | --- |
| BACH2 | Affinity Biosciences | DF2461 |
| BACH2 | Cell Signal Technology | 80775S |
| GRSF1 | Abcam | Ab205531 |
| p70 S6K | Cell Signal Technology | 2708S |
| p-p70 S6K (Thr421/Ser424) | Cell Signal Technology | 9204S |
| phosphor-Ser/Thr | Abcam | ab17464 |
| NF-κB | Cell Signal Technology | 8242S |
| p-NF-κB | Cell Signal Technology | 3033S |
| p-IκB | Abmart | TP70389 |
| NLRP3 | Cell signaling Technology | 15101S |
| NLRP3 | Novus Biologicals | NBP2-12446; |
| ASC | Cell signaling Technology | 67824S |
| CASPASE 1 | Cell signaling Technology | 24232S |
| CASPASE 1 | Proteintech | 22915-1-AP |
| Cleaved CASPASE 1 (p20) | Cell signaling Technology | 89332 |
| GSDMD | Abcam | ab209845 |
| GSDMD (N-terminal) | Affinity Biosciences | AF4013 |
| IL-1β | Cell signaling Technology | 31202S |
| IL-18 | Abcam | ab243091 |
| GAPDH | Cell signaling Technology | 5174S |
| β-Tubulin | Cell signaling Technology | 2128S |
| α-Actinin | Proteintech | 11313-2-AP |
| β-Actin | Santa Cruz Biotechnology | sc-47778 |
| LC-3 | Abcam | ab192890 |
| CASPASE 3 | Cell signaling Technology | 14220S |
| Cleaved CASPASE 3 | Cell signaling Technology | 9661S |
| BCL-2 | Cell signaling Technology | 3498S |
| BAX | Cell signaling Technology | 2772S |
| RIPK1 | Affinity | AF7877 |
| p-RIPK1 | Affinity | AF7377 |
| RIPK3 | Affinity | DF10141 |
| p-RIPK3 | Affinity | AF7422 |
| LAMIN B | Proteintech | 12987-1-AP |

**Table S5. Primers used in ChIP**

| Gene | Forward (5’-3’) | Reverse (5’-3’) |
| --- | --- | --- |
| *ASC-1* | TTAGTAGAGATGGGGTTTC | AACAAACAAGACAACCAGG |
| *ASC-2* | GGCCAGGCTGGTCTCAAAC | GCCACTCGGCAACAGACAC |
| *ASC-3* | GATCACCCACCTCGCCCTC | CTGCACTCTGTGCCACTCG |
| *ASC-4* | TGGTCTCAAACTCCTGATC | ATTGTGCCACTGCACTCTG |
| *ASC-5* | CTGGGATTACAGGCATGAG | TATGATTGTGCCACTGCAC |
| *NLRP3-1* | GTAGGTCTGTGTGTGTCTC | TCAAACTCCAGAGTCCATG |
| *NLRP3-2* | TCTCCTCAAGCTACTCAAG | AGTACAGGTTCTCTCCGAC |
| *NLRP3-3* | AAGCTGAGGCTTTGTGTGC | ACAGGTTCTCTCCGACATG |
| *NLRP3-4* | TGCCACATACCAGCCATTC | TACTCCCTGACTCATTGAC |
| *NLRP3-5* | ATTCCGTGAGTGTTAGTGG | ACAGGTTCTCTCCGACATG |

**Supplemental Figures**


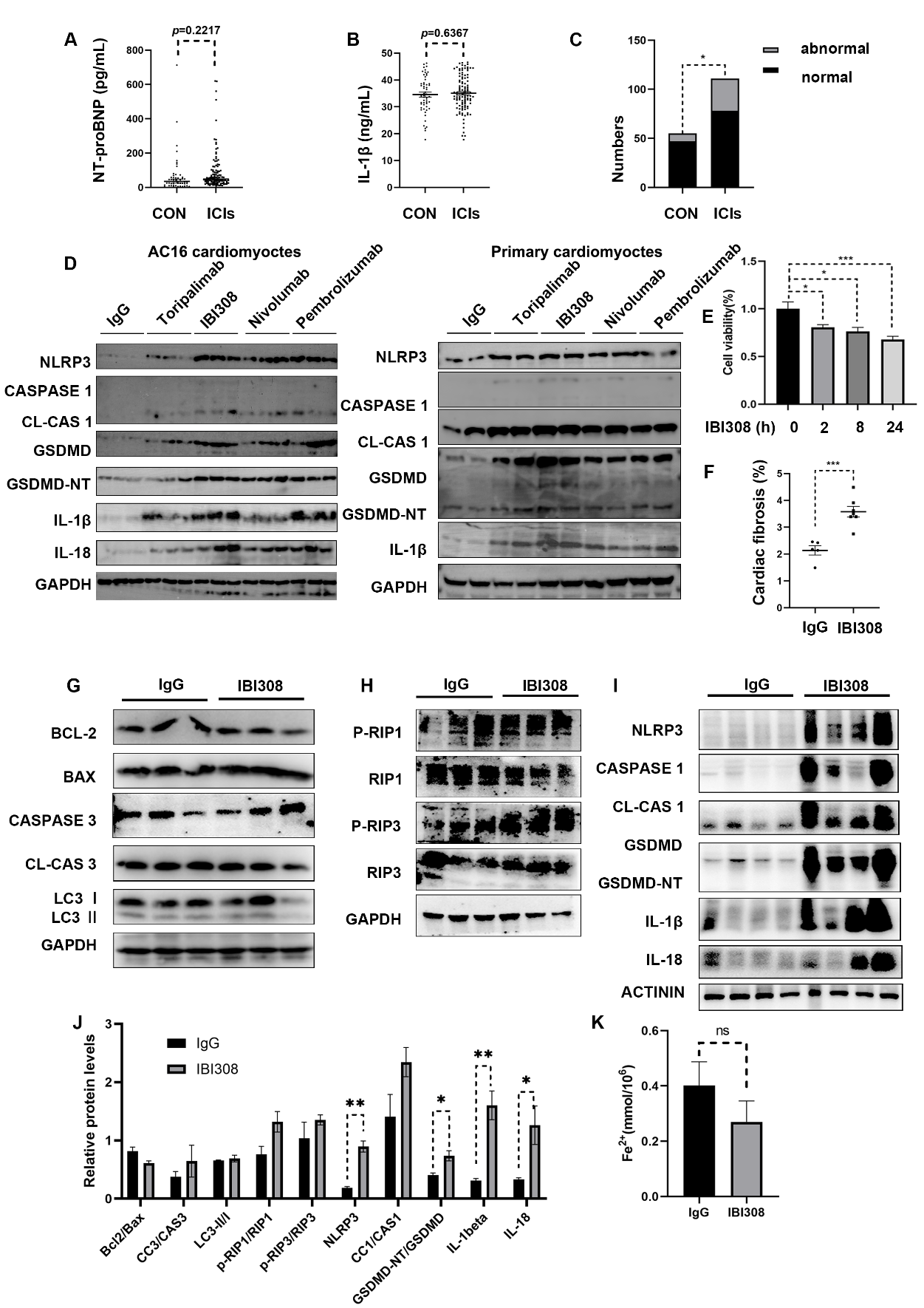
**Fig. S1. Immune checklist inhibitors (ICIs) induced myocyte injury and predominantly cell pyroptosis. (A and B)** The serum levels of NT-proBNP and IL-1β in clinical non-small cell lung cancer (NSCLC) patients with (ICIs group, n=111) or without (Control, or CON group, n=55) ICIs treatment. **(C)** Proportion of patients with cardiac abnormalities in the two clinical groups. **(D)**Western blot analysis of pyroptosis proteins in AC16 cardiomyocytes and primary cardiomyocytes incubated with or without four ICIs for 24 hours. **(E)** Cell viability in AC16 cardiomyocytes treated with IBI308 (4 μg/μL) for different hours. **(F)** Quantification of Masson Trichrome staining in 1J. **(G and H)** Western blot analysis of apoptosis, autophagy, and necroptosis in mouse hearts under IgG and IBI308 treatments (50 mg/kg, weekly for a month). **(I)** Western blot analysis of pyroptosis in mouse hearts under IgG and IBI308 treatments. **(J)** Quantification of apoptosis, autophagy, necroptosis and pyroptosis in mouse hearts under IgG and IBI308 treatments. **(K)** Concentration of Fe^2+^ in mouse hearts from each group. *, *p<*0.05; **, *p<*0.01; ***, *p<*0.001; ****, *p<*0.0001 as indicated.


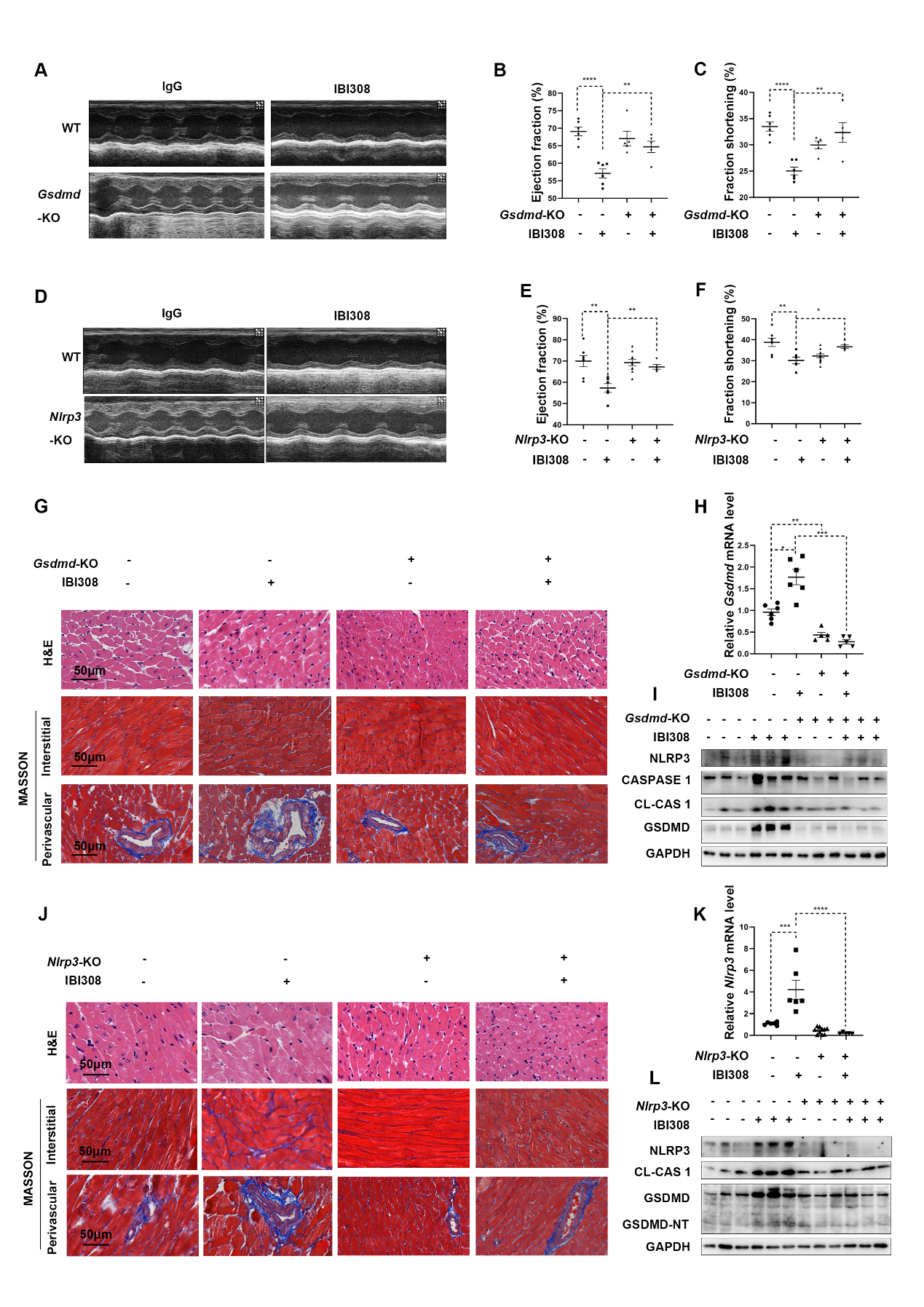


**Fig. S2. Knockout of *Gsdmd* or *Nlrp3* reverses the heart dysfunction induced by IBI308. (A)** Representative M­ mode echocardiographic images of *Gsdmd*-KO mice and WT mice with or without IBI308 treatment (50 mg/kg, weekly for a month) (n=5–6 per group). (**B and C**) Ejection fraction and fraction of shortening measurement of *Gsdmd*-KO mice and WT mice with or without IBI308 treatment (50 mg/kg, weekly for a month) (n=5–6 per group). **(D)** Representative M­ mode echocardiographic images of *Nlrp*3-KO mice and WT mice with or without IBI308 treatment (50 mg/kg, weekly for a month) (n=5–10 per group). (**E and F**) Ejection fraction and fraction of shortening measurement of *Nlrp3*-KO mice and WT mice (50 mg/kg, weekly for a month) (n=5–10 per group). **(G)** HE staining and Masson trichrome staining in *Gsdmd*-KO mice and WT mice hearts with indicated treatments. Scale bar= 50 μm. **(H)** Quantitative polymerase chain reaction analysis of the mRNA levels of *Gsdmd* in the heart of mice from each group. **(I)** Western blot analysis of the NLRP3-associated pyroptosis in the heart of *Gsdmd*-KO mice and WT mice under IgG or IBI308 treatments. **(J)** HE staining and Masson trichrome staining in *Nlrp*3-KO mice and WT mice hearts with indicated treatments. **(K)** Quantitative polymerase chain reaction analysis of the mRNA levels of *Nlrp*3 in the heart of each mouse. **(L)** Western blot analysis of the NLRP3-associated pyroptosis in the heart of *Nlrp*3-KO mice and WT mice under IgG or IBI308 treatments. *, *p<*0.05; **, *p<*0.01; ***, *p<*0.001; ****, *p<*0.0001 as indicated.


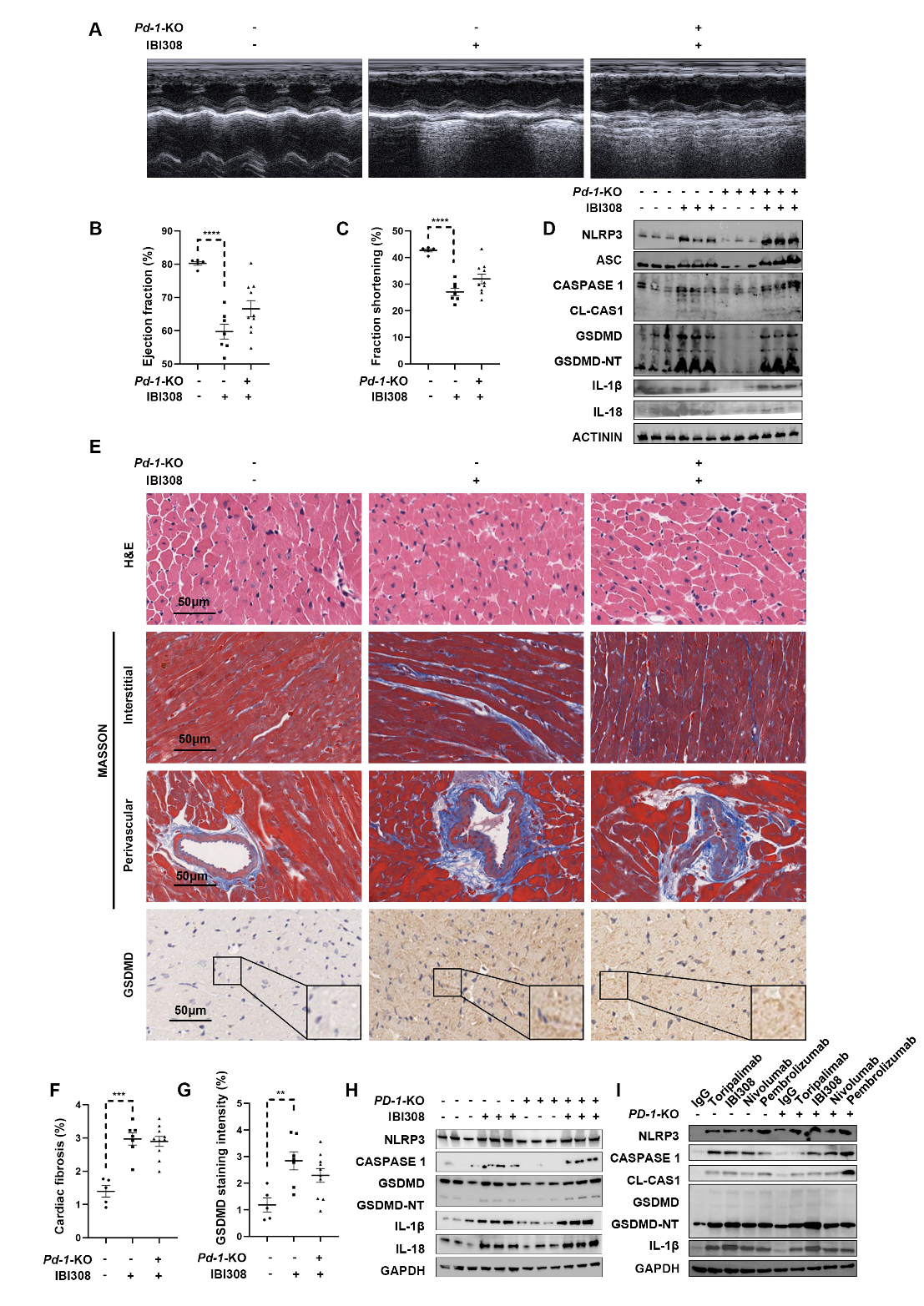


**Fig. S3. Effects of IBI308 on hearts from *Pd-1*-KO mice. (A)** Representative M­ mode echocardiographic images of *Pd-1*-KO mice and WT mice with or without IBI308 treatment (50 mg/kg, weekly for a month) (n=5–10 per group). **(B and C)** Ejection fraction (EF) and fraction of shortening (FS) of each mouse. **(D)** Western blot analysis of pyroptosis in the mice hearts. **(E)** HE staining, Masson trichrome staining and immunohistochemistry staining of GSDMD from each mouse. Scale bar= 50 μm. **(F)** Quantification of cardiac fibrosis from Masson Trichrome staining. **(G)** Quantification of GSDMD staining intensity. **(H)** Western blot analysis of the NLRP3-associated pyroptosis in PD-1-KO cells and control cells with or without IBI308 treatment (4 μg/μL) for 24 hours. **(I)** Western blot analysis of the NLRP3-associated pyroptosis in PD-1-KO cells and control cells with or without treatment of Toripalimab, IBI308, Nivolumab and Pembrolizumab. *, *p<*0.05; **, *p<*0.01; ***, *p<*0.001; ****, *p<*0.0001 as indicated.


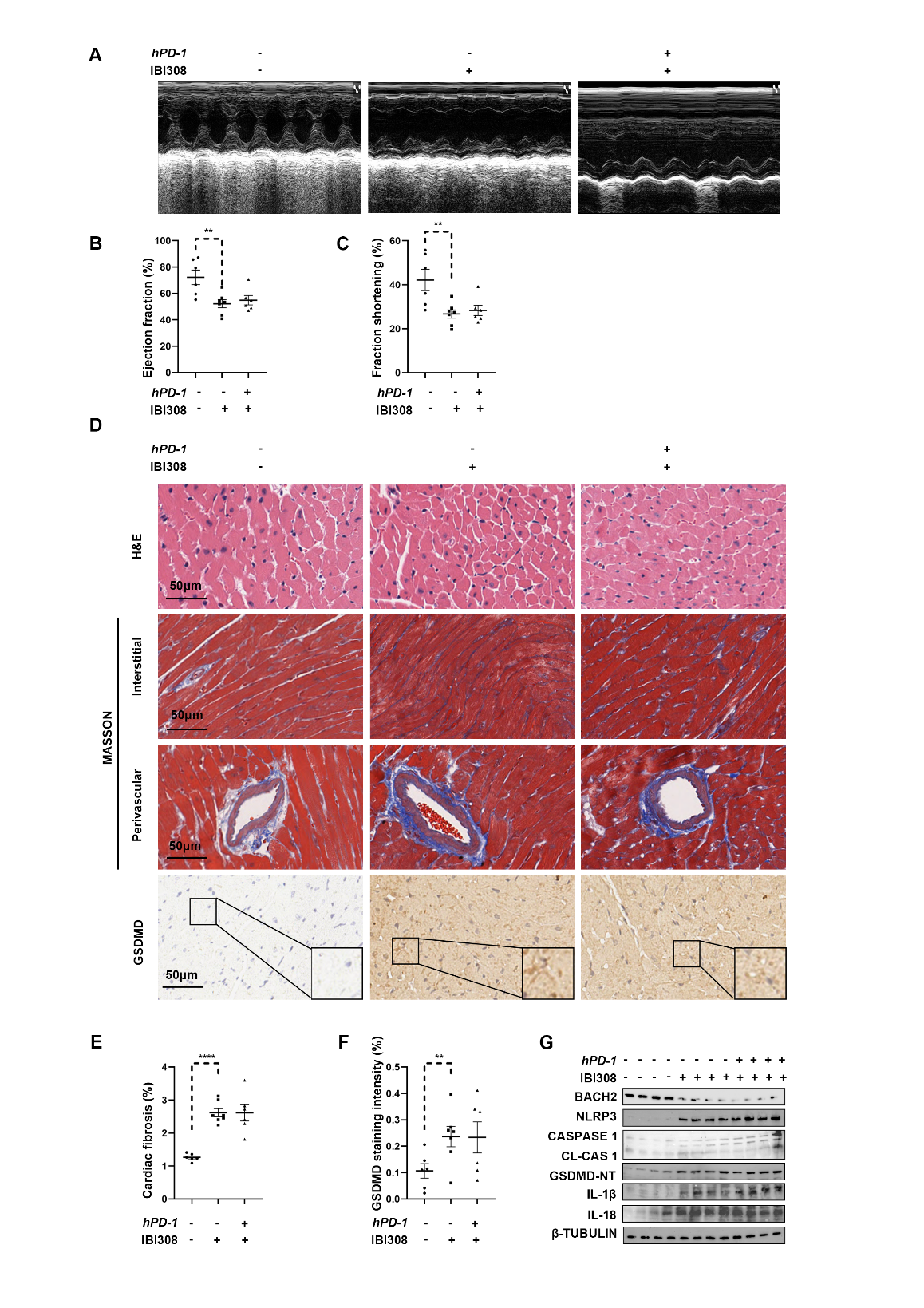


**Fig. S4. Effects of IBI308 on hearts from humanized *PD-1* mice (*hPD-1*). (A)** Representative M­ mode echocardiographic images of *hPD-1* mice and WT mice with or without IBI308 treatment (50 mg/kg, weekly for a month) (n=6–7 per group). **(B and C)** Ejection fraction (EF) and fraction of shortening (FS) of each mouse. **(D)** HE staining, Masson trichrome staining and immunohistochemistry staining of GSDMD from each mouse. Scale bar= 50 μm. **(E)** Quantification of cardiac fibrosis from Masson Trichrome staining. **(F)** Quantification of GSDMD staining intensity. **(G)** Western blot analysis of BACH2 and pyroptosis in the mice hearts. *, *p<*0.05; **, *p<*0.01; ***, *p<*0.001; ****, *p<*0.0001 as indicated.

**
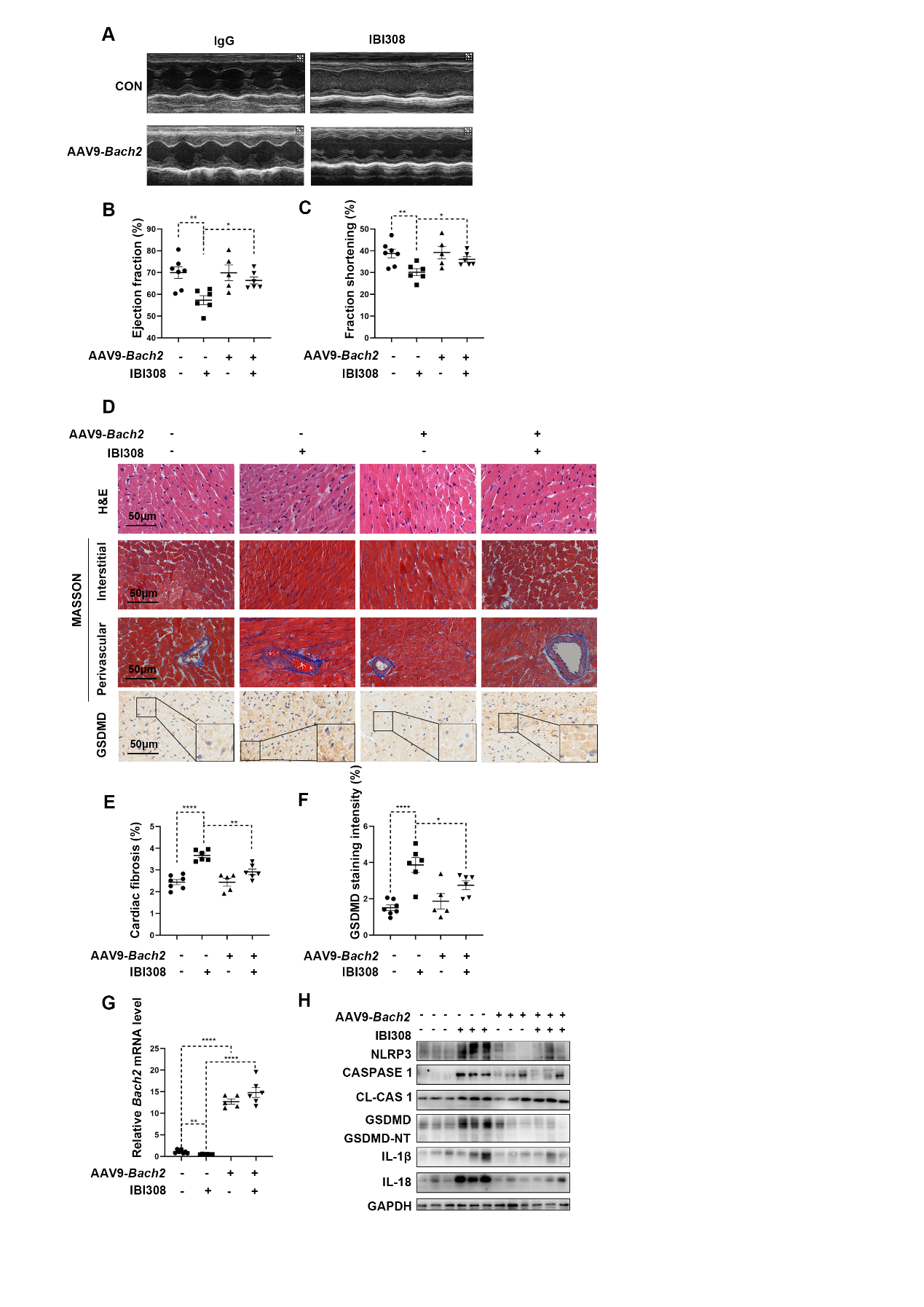
Fig. S5. AAV9-mediated overexpression of BACH2 relieves IBI308-induced cardiotoxicity.** Mice were injected with Bach2-delivering adeno-associated virus 9 (AAV9- Bach2) or control virus (AAV9-Con) through tail vein, followed by treatment of IgG or IBI308 (50 mg/kg, weekly for a month) (n=5–7 per group). **(A)** M­ mode echocardiography was conducted and representative images were presented. **(B and C)** Ejection faction (EF) and faction of shortening (FS) of each mouse. **(D)** HE staining, Masson trichrome staining and immunohistochemistry staining of GSDMD from each mouse. Scale bar= 50 μm. **(E)** Quantification of cardiac fibrosis from Masson Trichrome staining. **(F)** Quantification of GSDMD staining intensity. **(G)** Relative mRNA levels of *Bach2*. **(H)** Western blot analysis of pyroptosis in the mice hearts. *, *p<*0.05; **, *p<*0.01; ****, *p<*0.0001 as indicated.


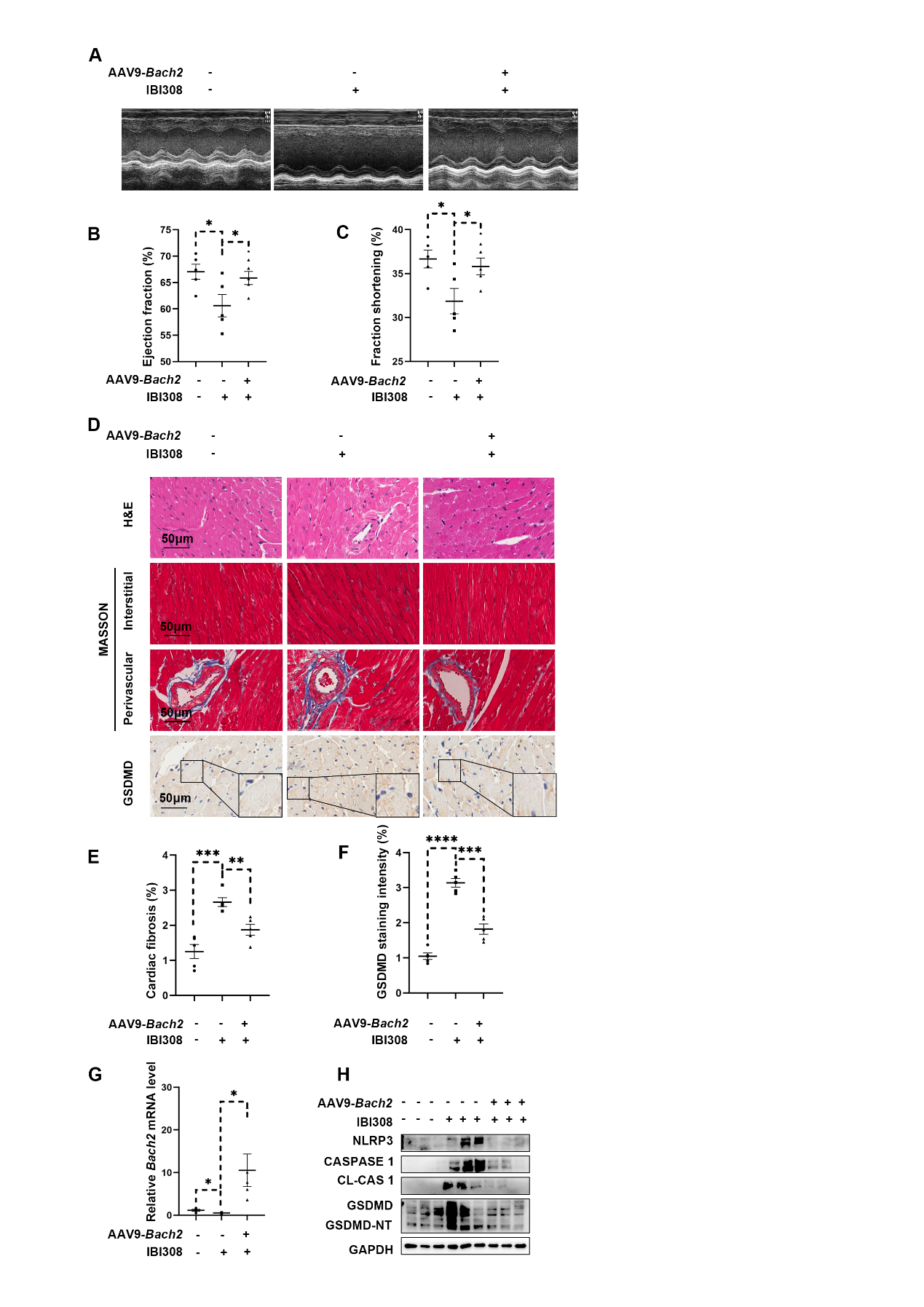


**Fig. S6. Bach2 protected against IBI308 cardiotoxicity in nude mice.** Nude mice were injected with Bach2-delivering adeno-associated virus 9 (AAV9- Bach2) or control virus (AAV9-Con) through tail vein, followed by treatment of IgG or IBI308 (50 mg/kg, weekly for a month) (n=5 per group). **(A)** M­ mode echocardiography was conducted and representative images were presented. **(B and C)** Ejection fraction (EF) and fraction of shortening (FS) of each mouse. **(D)** HE staining, Masson trichrome staining and immunohistochemistry staining of GSDMD from each mouse. Scale bar= 50 μm. **(E)** Quantification of cardiac fibrosis from Masson Trichrome staining. **(F)** Quantification of GSDMD staining intensity. **(G)** Relative mRNA levels of *Bach2*. **(H)** Western blot analysis of pyroptosis in the mice hearts. *, *p<*0.05; **, *p<*0.01; ***, *p<*0.001; ****, *p<*0.0001 as indicated.


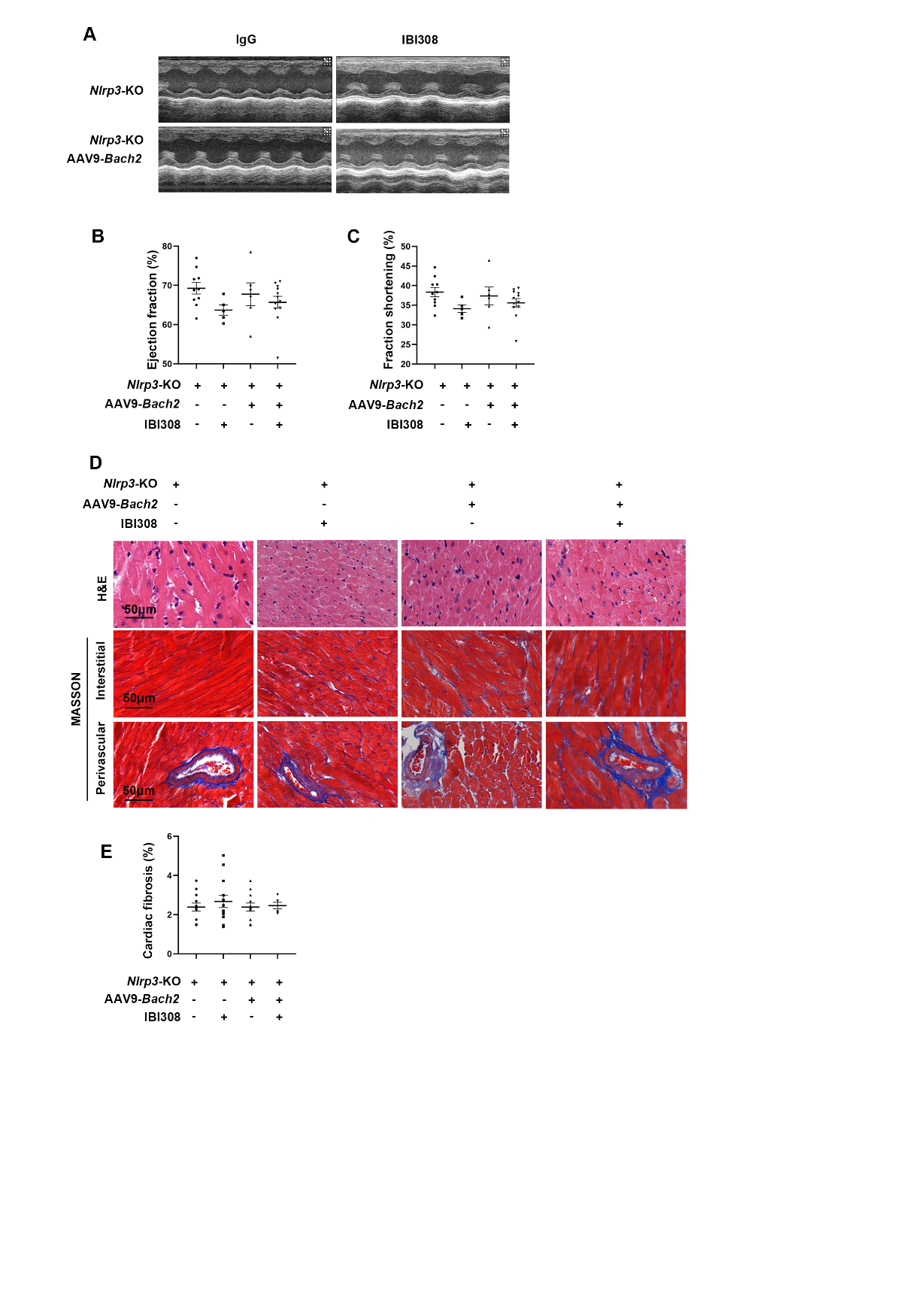


**Fig. S7. Protective effects of BACH2 were dampened in *Nlrp3-KO* mice.** *Nlrp*3-KO mice were injected with Bach2-delivering adeno-associated virus 9 (AAV9- Bach2) or control virus (AAV9-Con) through tail vein, followed by treatment of IgG or IBI308 (50 mg/kg, weekly for a month) (n=5-12 per group). **(A)** M­ mode echocardiography was conducted and representative images were presented. **(B and C)** Ejection fraction (EF) and fraction of shortening (FS) of each mouse. **(D)** HE staining and Masson Trichrome staining from each group. Scale bar= 50 μm. **(E)** Quantification of cardiac fibrosis from the slides after Masson Trichrome staining.


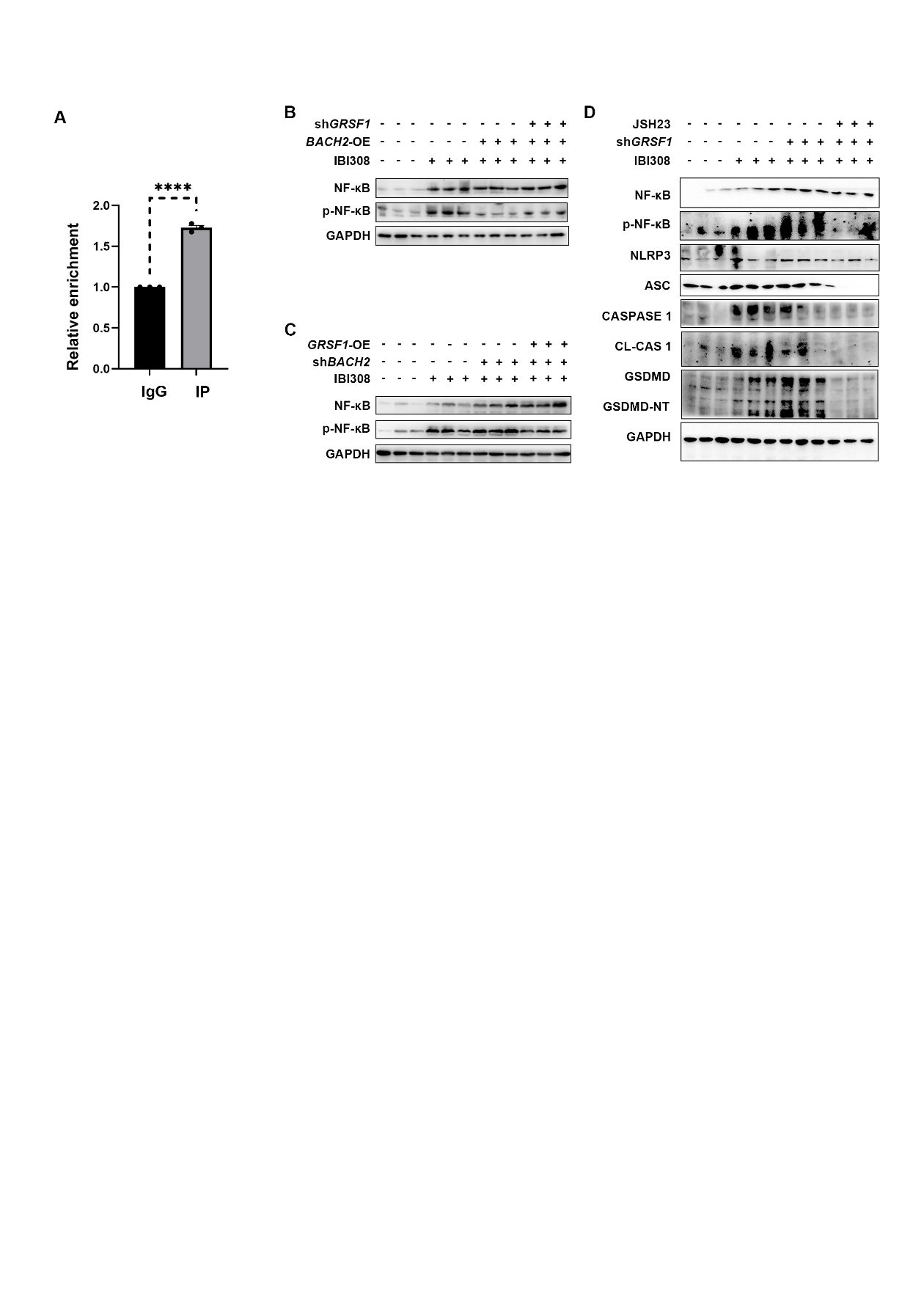


**Fig. S8. BACH2/GRSF1 protected against IBI308-induced myocyte pyroptosis via NF-κB pathway. (A)** Relative enrichment of Nfκbib in RIP assays. **(B and C)** Western blot analysis of NF-κB and its phosphorylation in AC16 cells with indicated treatments. IBI308=4 μg/μL for 24 hours. **(D)** Western blot analysis of NF-κB signaling and pyroptosis in AC16 cells with indicated treatments. IBI308=4 μg/μL, and JSH23=3 μM for 24 hours. ****, *p<*0.0001.


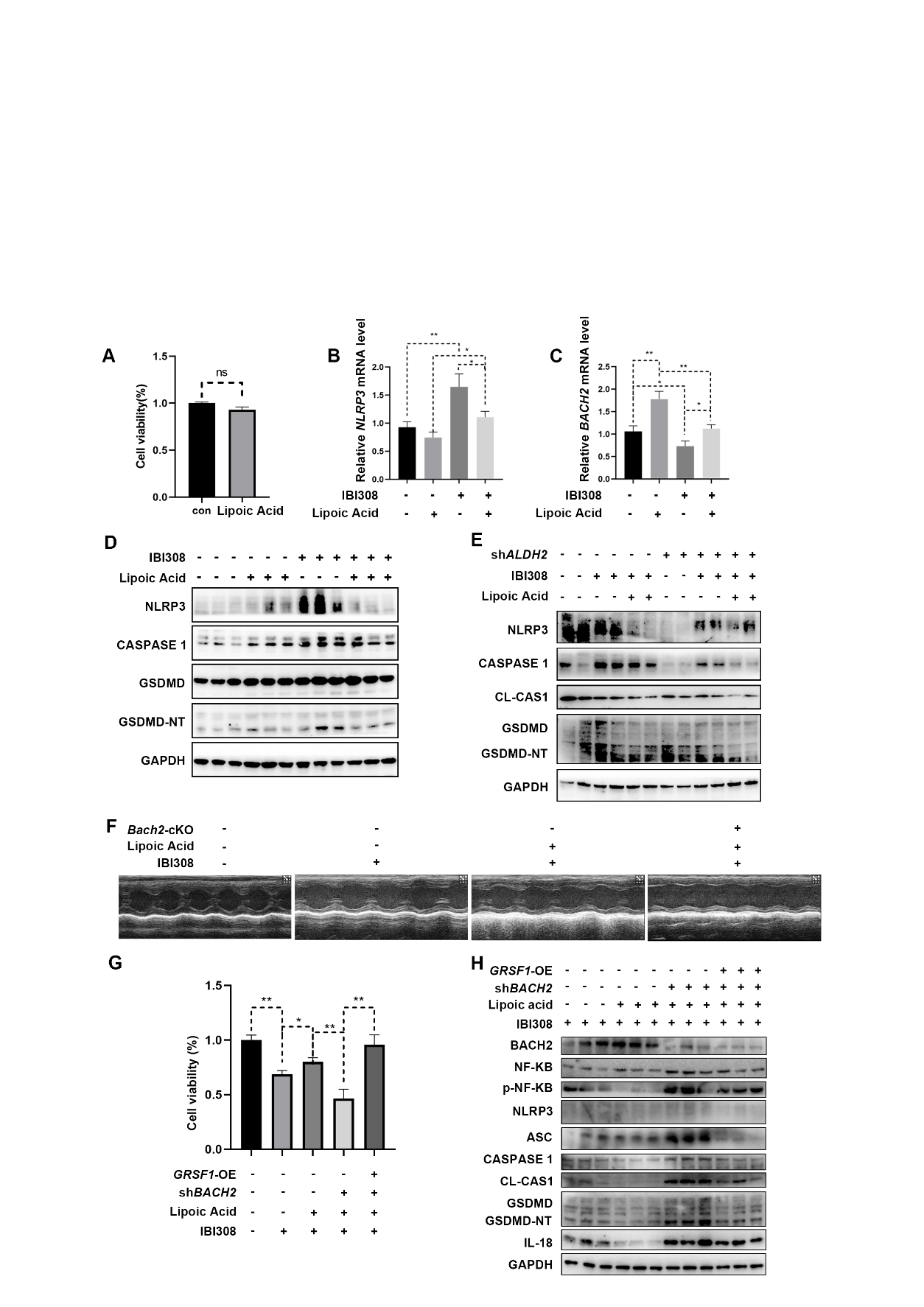


**Fig. S9. Lipoic acid attenuates IBI308-induced pyroptosis. (A)** Cell viability of AC16 cells treated with lipoic acid. **(B and C)** Relative mRNA levels of *NLRP3* and BACH2 in AC16 cells treated with IBI308 (4 μg/μl) and lipoic acid (10 μM) for 24 hours as indicated. **(D)** Western blot analysis of pyroptosis in AC16 cells treated with IBI308 (4 μg/μl) and lipoic acid (10 μM) for 24 hours as indicated. **(E)** Western blot analysis of pyroptosis when sh*NC* and sh*ALDH2* AC16 cells were treated with IgG and IBI308 (4 μg/μl) for 24 hours, with or without lipoic acid (10 μM). **(F)** Lipoic acid (4mg/kg, daily for five weeks) was monitored into *Bach2*-cKO mice and WT mice in the presence or absence of IBI308 stimulation (50 mg/kg, weekly for a month) (n=7–9 per group). M­ mode echocardiography was conducted and representative images were presented. **(G)** Cell viability of AC16 cells when BACH2 and GRSF1 were modulated, and IBI308 (4 μg/μl) and lipoic acid (10 μM) were added as indicated for 24 hours. **(H)** Western blot analysis of BACH2, NF-κB pathway and pyroptosis in AC16 cells. *, *p<*0.05; **, *p<*0.01 as indicated.
